# Hydrogen and Lithium Bonds—Lewis Acid Units Possessing Multi-Center Covalent Bonds

**DOI:** 10.3390/molecules26226939

**Published:** 2021-11-17

**Authors:** Mohammad Aarabi, Samira Gholami, Sławomir J. Grabowski

**Affiliations:** 1 Dipartimento di Chimica Industriale “Toso Montanari”, Università degli Studi di Bologna, Viale del Risorgimento 4, I-40136 Bologna, Italy; mohammad.aarabi@unibo.it (M.A.); samira.gholami2@unibo.it (S.G.); 2 Polimero eta Material Aurreratuak: Fisika, Kimika eta Teknologia, Kimika Fakultatea, Euskal Herriko Unibertsitatea UPV/EHU & Donostia International Physics Center (DIPC) PK 1072, 20080 Donostia, Euskadi, Spain; 3 IKERBASQUE, Basque Foundation for Science, 48011 Bilbao, Spain

**Keywords:** hydrogen bond, lithium bond, multi-center covalent bond, QTAIM, SAPT, NCI method

## Abstract

MP2/aug-cc-pVTZ calculations were carried out on complexes wherein the proton or the lithium cation is located between π-electron systems, or between π-electron and σ-electron units. The acetylene or its fluorine and lithium derivatives act as the Lewis base π-electron species similarly to molecular hydrogen, which acts as the electron donor via its σ-electrons. These complexes may be classified as linked by π-H∙∙∙π/σ hydrogen bonds and π-Li∙∙∙π/σ lithium bonds. The properties of these interactions are discussed, and particularly the Lewis acid units are analyzed, because multi-center π-H or π-Li covalent bonds may occur in these systems. Various theoretical approaches were applied here to analyze the above-mentioned interactions—the Quantum Theory of Atoms in Molecules (QTAIM), the Symmetry-Adapted Perturbation Theory (SAPT) and the Non-Covalent Interaction (NCI) method.

## 1. Introduction

There are three issues connected with the physical meaning of chemical bonds, and consequently with their definition:

(1) More than one electron pair may be located between two atomic centers, thus double and triple bonds are observed. This issue was taken into account by Butlerow in his studies on structural chemistry [1];

(2) Some intra- and intermolecular contacts (interactions) may possess characteristics of covalent bonds. This was widely analyzed and commented upon in relation to hydrogen bond interactions, since their covalency or partly covalent character were discussed [2];

(3) According to the Lewis definition of the chemical bond, it is a strong two-center–two-electrons interaction, 2c/2e. However, it seems that multi-center bonds also exist, such as three-center ones [3].

The above issues are discussed in this study for the complexes analyzed. Let us refer to the issue concerning three-center bonds. Such systems were described in detail by Weinhold and Landis [3]. The 3c/4e (three-center–four-electron) term is related to hypervalency, where the octet rule is not obeyed and the center considered is characterized by the excess of electrons. It concerns three-center ω-bonds, and this kind of arrangement was discussed recently for various types of interactions [4].

The 3c/2e bonds (three-center–two-electron) also concern centers not obeying the octet rule; however, in this case, electron deficiency occurs at the center considered, and this is known as hypovalency. In this case, “three-center character can alternatively be achieved by a two-center donor interacting with a one-center acceptor (e.g., σ_AC_ → n_B_*). In this case the three starting valence hybrids (h_A_, h_B_, and h_C_) are occupied only by the two electrons of the two-center donor.” [3]. The corresponding 3c/2e bonds are designated as τ_ABC_. Such arrangements occur often in boron hydrides (τ_BHB_), and have been discussed for protonated ethylene, C_2_H_4_∙∙∙H^+^ (τ_CHC_), and the H_3_^+^ cation (τ_HHH_) [3].

There are other examples of 3c/2e systems. The theoretical analysis was performed in an early study on the following species: C_2_H_4_∙∙∙Cu^+^, C_2_H_4_∙∙∙Ag^+^, and C_2_H_4_∙∙∙Au^+^. The Hartree–Fock calculations, using the Slater-type basis sets, show dissociation energies in these systems of about 50–70 kcal/mol [5]. These energies are mainly electrostatic, but a significant part is related to electron charge shifts between the coinage cation and the ethylene species. One can see that the occurrence of τ_CMC_ (M = Cu, Ag or Au) 3c/2e bonds may be considered for these systems. More accurate DFT calculations (BP86/TZP method, Slater-type basis sets) were performed later for the same systems and for their acetylene analogues, C_2_H_2_∙∙∙M^+^ [6]. The decomposition of interaction energies was also performed for these systems, and it was found that the electrostatic contribution is the most important part of the stabilization energy, comprising almost 60% of all attractive interaction energy terms. However, the orbital interaction energy is also very important, as it covers slightly more than 40% of the attractive energy [6]. The dissociation energies for these species, between 35 and 80 kcal/mol, show that the M∙∙∙CC contacts may be considered as τ_CMC_ multi-center covalent bonds.

For the C_2_H_2_∙∙∙H^+^ species, two conformations occur that correspond to local energetic minima. The T-shaped conformation is characterized by lower energy than other conformations, and it may act as a proton donor in the π-H∙∙∙π hydrogen bonds. This kind of interaction was found in the C_2_H_4_-H^+^∙∙∙C_2_H_2_ and C_2_H_2_-H^+^∙∙∙C_2_H_2_ complexes, which were analyzed theoretically, since calculations up to the MP2/6-311++G(3d,3p) level have already been performed for them [7]. It was found that the proton in these complexes is located closer to one of the π-electron systems forming the multi-center π-H covalent bond. The other H∙∙∙π interactions in these systems are weaker than π-H ones, but still strong, and they possess a partly covalent character. It is interesting that the existence of a C_2_H_2_-H^+^∙∙∙C_2_H_2_ complex was confirmed experimentally by the infrared photodissociation spectroscopy [8].

Similar systems to those mentioned above here are analyzed from time to time; more recently, complexes with H^+^, Au^+^ and Li^+^ cations situated between π-electron species have been analyzed theoretically [9]. One can also mention the acetylene complexes of the Ag^+^ cation, where larger clusters containing more than two acetylene molecules were analyzed [10].

The aim of this study is to analyze the properties of the 3c/2e bonds that form between the H^+^ or Li^+^ cation and the acetylene molecule or its simple derivative. The influence of lithium and fluorine substituents on the Lewis base properties of acetylene is also discussed in this study. However, interactions in complexes where the above-mentioned proton or lithium cation is situated between π-electron systems or between π-electron and σ-electron systems are analyzed in particular. These are special kinds of hydrogen and lithium bonds.

Various theoretical approaches are applied here, including the Quantum Theory of Atoms in Molecules (QTAIM) [11,12], the Symmetry-Adapted Perturbation Theory (SAPT) [13] and the Non-Covalent Interaction (NCI) method [14,15], and standard theoretical analyses of the interaction and binding energies [16] and of the electron charge distributions are performed here as well [17,18,19].

## 2. Computational Methods

The calculations were performed with the Gaussian16 set of codes [20]. The MP2 method [21] and the Dunning-style aug-cc-pVTZ basis set [22,23] were applied to perform optimizations of complexes analyzed in this study. Frequency calculations have been carried out at the same level to confirm that the structures optimized correspond to energetic minima. The following complexes are analyzed in this study: those with the proton inserted between π-electron systems; acetylene, C_2_H_2_, or its derivatives (the FCCF and LiCCLi species are considered as such systems). These complexes are connected by the π-H∙∙∙π interactions. The counterparts of the above complexes are also analyzed where the lithium cation, Li^+^, is located between π-electron systems. They are connected by π-Li∙∙∙π interactions. The next group of complexes are characterized by the proton or lithium cation being inserted between the above-mentioned acetylene or its derivative and the dihydrogen. These complexes are linked by the π-H∙∙∙σ or π-Li∙∙∙σ interactions.

**Figure 1:**
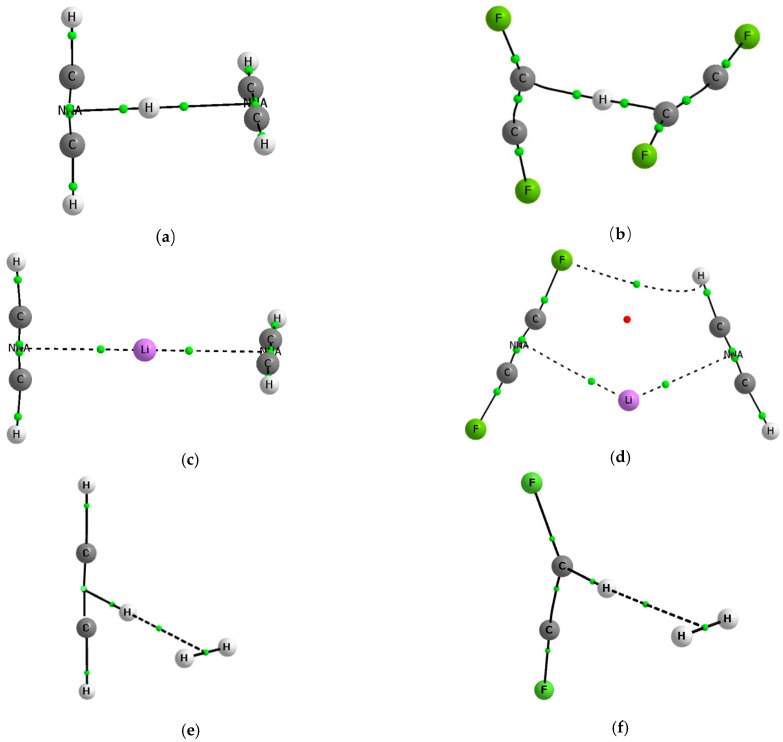

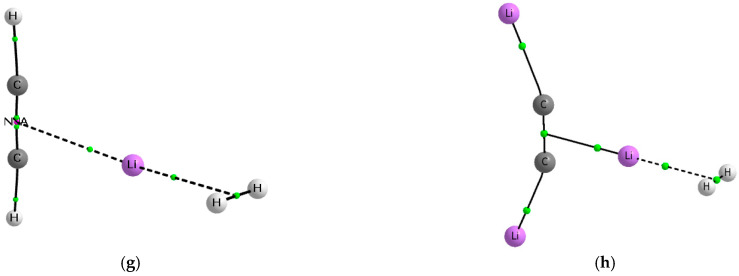
Molecular graphs of selected complexes analyzed in this study; (**a**) C_2_H_3_^+^-C_2_H_2_, (**b**) C_2_F_2_H^+^-C_2_F_2_, (**c**) C_2_H_2_Li^+^-C_2_H_2_, (**d**) C_2_H_2_Li^+^-C_2_F_2_, (**e**) C_2_H_3_^+^-H_2_, (**f**) C_2_F_2_H^+^-H_2_, (**g**) C_2_H_2_Li^+^-H_2_, (**h**) C_2_Li_3_^+^-H_2_. Big circles correspond to attractors, small green circles to bond critical points, small red circles to ring critical point (in (**d**), the C_2_H_2_Li^+^-C_2_F_2_ complex) and to non-nuclear attractors (NNAs). The continuous and broken lines correspond to bond paths.

Figure 1 presents molecular graphs of selected complexes (molecular graphs of all complexes investigated in this study are presented in Supplementary Information, Figure S1). For two complexes, C_2_Li_2_-H^+^-C_2_Li_2_ and C_2_Li_2_-H^+^-C_2_H_2_, optimizations lead to structures with the H^+^ cation located outside the π∙∙∙π area. Such complexes are not a subject of this study.

The interaction and binding energies, E_int_ and E_bin_, respectively, are calculated here. It is assumed that the complexes are built from two monomers: the dihydrogen or acetylene system and the acetylene system with the H^+^ or Li^+^ cation attached; this division is explained in detail in the next section.

In general, the interaction energy of the A…B complex composed of A and B monomers is calculated according to the supermolecular approach (Equation (1)) [16].

E_int_ = E_A…B_(A…B) − E_A…B_(A) − E_A…B_(B) (1)

The symbols in parentheses correspond to systems for which energies are calculated, the subscripts inform the geometry optimized. This means that the interaction energy is the difference between the energy of the A…B complex and the energies of A and B species. The monomers are characterized by the geometries that they have in the complex. The binding energy takes into account the deformation energy (E_def_), and it has the following form [24]:

E_bin_ = E_int_ + E_def_ = E_A…B_(A…B) − E_A_(A) − E_B_(B) (2)

For the binding energy (Equation (2)), the energies of separately optimized monomers are considered. The deformation energy (Equation (3)) is positive, since monomers with geometries taken from the complex are not in energetic minima [16,24].

E_def_ = E_A…B_(A) + E_A…B_(B) − E_A_(A) − E_B_(B) (3)

The E_int_ and E_bin_ energies corrected for the Basis Set Superposition Error (BSSE) [25] are discussed further here.

The QTAIM approach [11,12] was applied to analyze characteristics of bond critical points (BCPs) corresponding to bond paths between the hydrogen or lithium center of a complex and the neighbouring species. The QTAIM charges that result from integrations over corresponding basins are also discussed in this study. The AIMAll program [26] was used to perform QTAIM calculations.

The SAPT method [13] was applied to calculate the terms of energies of interactions for the complexes analyzed; the Psi4 program was used to perform the calculations [27]. The SAPT calculations performed with the use of the aug-cc-pVTZ basis set correspond to the optimizations at the MP2/aug-cc-pVTZ-level geometries. The SAPT is an approach used to calculate the interaction energy of two closed-shell moieties, which is obtained directly as a sum of defined terms. Hence it differs from the other commonly applied approaches (including decomposition schemes), wherein the interaction energy is calculated as the difference between the energy of the complex and the sum of energies of monomers.

The interaction energy in the SAPT approach is the sum of the following terms: the first-order electrostatics (*E*_elst_^(1)^), second-order induction (*E*_ind_^(2)^) and dispersion (*E*_disp_^(2)^) energies, and their exchange counterparts (first-order exchange (*E*_exch_^(1)^), second-order exchange-induction (*E*_exch-ind_^(2)^) and exchange-dispersion (*E*_exch-disp_^(2)^)). Up to the second order, the SAPT approach contains a main part of the energy of interaction. To take into account the higher-order induction and exchange-induction energies, the Hartree–Fock “delta” correction term *δE*_HF_ is included. Hence the so-called SAPT2 interaction energy is calculated according to Equation (4).

(4) $$E_{int}^{SAPT2}=E_{\mathrm{exch}}{}^{\left(1\right)}+E_{\mathrm{elst}}{}^{\left(1\right)}+E_{\mathrm{ind}}{}^{\left(2\right)}+E_{\mathrm{disp}}{}^{\left(2\right)}+E{}_{\mathrm{exch}-\mathrm{ind}}{}^{\left(2\right)}+E{}_{\mathrm{exch}-\mathrm{disp}}{}^{\left(2\right)}+{\delta E}_{\mathrm{HF}}\;$$

In the truncation of the SAPT energy that is employed in this study, the following groupings of energy contributions may be performed (Equation (5)):

(5) $$E_{int}^{SAPT2}=E_{\mathrm{exch}}+E_{\mathrm{elst}}+E_{\mathrm{ind}}+E_{\mathrm{disp}}$$

where *E*_exch_ = *E*_exch_^(1)^, *E*_elst_ = *E*_elst_^(1)^, *E*_ind_ = *E*_ind_^(2)^ + *E*_exch-ind_^(2)^ + *δE*_HF_, and *E*_disp_ = *E*_disp_^(2)^ + *E*_exch-disp_^(2)^ [28].

The NCI method has also been applied in this study to analyze interactions in complexes discussed [14,15,29]. These calculations have been carried out with the use of the MultiWFN program [30]. The reduced density gradient (RDG) isosurfaces and the corresponding scatter graphs were visualized by VMD 1.9.3 and OriginPro 2016 packages, respectively [31,32].

## 3. Results and Discussion

### 3.1. Geometries

**Scheme 1:**
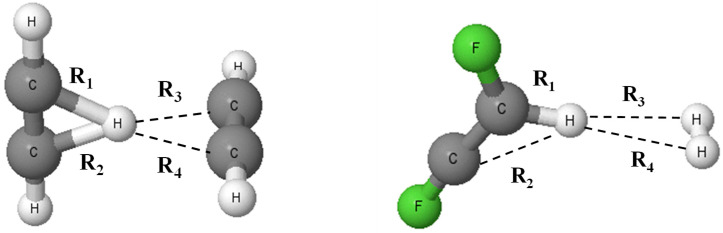
The definition of distances presented in Table 1.

Table 1 presents the distances between the proton or the lithium cation and the carbon or hydrogen atoms of neighbouring species (Scheme 1). The complexes in Table 1 and other tables are marked as follows: the H^+^ or Li^+^ cation is connected with the closer system if it is located between π-electron species. If this cation is situated between the same species, it is assumed that it is attached to one of them, regardless of geometry. For example, for the C_2_H_3_^+^-C_2_H_2_ complex (Figure 1a), the proton is closer to one of the acetylene molecules, but for the C_2_H_2_Li^+^-C_2_H_2_ complex (Figure 1c), the Li^+^ cation is in the mid-point between acetylene molecules. In the case of dihydrogen complexes, the H^+^ or Li^+^ cation is attached to the π-electron system in spite of distances. This is justified since the cation interacts more strongly with the π-electron system than with the σ-electron one [33].

Four complexes with the proton between π-electron systems are analyzed here. In C_2_H_3_^+^-C_2_H_2_ (Figure 1a), C_2_H_3_^+^-C_2_F_2_ and C_2_Li_2_H^+^-C_2_F_2_ complexes, the proton is closer to one of the acetylene species. The C_2_H_3_^+^-C_2_H_2_ complex was analyzed earlier [7], and it was found that the proton is closer to one of the acetylene molecules forming the multi-center covalent bond, while the interaction of the proton with the further acetylene molecule is weaker, but it is still strong and partly covalent in nature. The π-H∙∙∙π link possesses numerous characteristics of a hydrogen bond [7]. Proton transfer between two acetylene molecules is possible, and for the transition state of this process, the proton is located in the mid-point of the acetylene–acetylene contact. The potential barrier height for the proton transfer amounts to only ~0.04 kcal/mol [8]. The asymmetry of the H-center position in the C_2_H_3_^+^-C_2_H_2_ complex was also confirmed by the CASPT2/aug-cc-pVTZ calculations, which also indicate that the nondynamic correlation for this system is not important [9].

In the C_2_F_2_H^+^-C_2_F_2_ complex (Figure 1b), the proton is located between two carbon atoms of the C_2_F_2_ units, with equivalent short C···H distances amounting to 1.394 Å. This means that the H-atom between two carbon atoms possesses the characteristics of a divalent center. The divalent character of the H-center was discussed earlier for the FHF^−^ anion by Pauling [34], and later, this anion, was analyzed in numerous experimental and theoretical studies [35,36,37].

Table 1 also presents the distances between the proton and the carbon centers for the isolated C_2_H_3_^+^, C_2_Li_2_H^+^ and C_2_F_2_H^+^ species. These distances increase if the latter cations interact with the additional π-electron or σ-electron system. This increase is much less pronounced in complexes with dihydrogen. For the C_2_F_2_H^+^ species, the proton is closer to one of the carbon atoms, practically forming a covalent bond, since the C∙∙∙H distance amounts to 1.095 Å. The C_2_F_2_H^+^ structure is very similar to the C_2_H_3_^+^ vinyl cation structure. The latter system is characterized by higher energy than other T-shaped conformations. Table 1 shows that the short C∙∙∙H distance of the C_2_F_2_H^+^ cation is elongated in complexes with the C_2_F_2_ species (Figure 1b) and with dihydrogen (Figure 1f)—the C-H∙∙∙C and C-H∙∙∙σ hydrogen bonds are observed for these complexes, respectively. The remaining complexes with the proton located between π-electron systems are linked by the π-H∙∙∙π hydrogen bonds, and those with the proton between π-electron and σ-electron systems by the π-H∙∙∙σ hydrogen bonds.

Let us consider the geometry of lithium cation species. The C_2_H_2_Li^+^, C_2_F_2_Li^+^, and C_2_Li_3_^+^ geometries are only slightly changed by additional interactions with the π-electron and σ-electron systems. The Li^+^ cation in such complexes is not moved significantly from the prime π-electron species; the same is not true for the proton-bound complexes, where the complexation moves the proton away from the prime acetylene or acetylene-like system.

### 3.2. Energies

The interaction and binding energies, E_int_ and E_bin_, respectively [16], as well as the deformation energy, E_def_ [24], and the basis set superposition error correction, BSSE [25], of complexes analyzed here are presented in Table 2. The BSSE correction usually does not exceed 1 kcal/mol; it is greater only for two complexes linked by the π-H∙∙∙π interaction, and for the C_2_F_2_H^+^-C_2_F_2_ complex, where the greatest BSSE correction of 1.9 kcal/mol is observed.

The E_int_ and E_bin_ are related to the division of complexes into monomers that was discussed earlier (in tables, monomers of complexes are separated by a dash). For greater changes in the geometries of monomers resulting from complexation, greater E_def_ values are observed. The greatest E_def_ is observed for the C_2_F_2_H^+^-C_2_F_2_ complex (Figure 1b), equal to 23.9 kcal/mol. Only in three other complexes is a meaningful deformation energy observed; for C_2_H_3_^+^-C_2_H_2_, C_2_H_3_^+^-C_2_F_2_ and C_2_Li_3_^+^-C_2_Li_2_ it amounts to 4.3, 2.4 and 2.4 kcal/mol, respectively. For the remaining complexes, the E_def_ value does not exceed 0.3 kcal/mol.

Strong interactions are observed for complexes connected by the π-H∙∙∙π and π-Li∙∙∙π links. The strongest interaction is observed for the C_2_Li_3_^+^-C_2_Li_2_ system, where the E_int_ value amounts to −52.0 kcal/mol. Next, for the C_2_F_2_H^+^-C_2_F_2_ complex, E_int_ is equal to −29.4 kcal/mol. For the latter complex, a high deformation energy is observed, thus the binding energy, E_bin_, is equal to −5.6 kcal/mol. The C_2_Li_2_H^+^-C_2_F_2_ complex is the only one with a H^+^ or Li^+^ cation between π-electron systems characterized by a weak interaction, where the E_int_ is equal to −1.5 kcal/mol only. Such a weak interaction may be explained in the following way. The CC bond of the C_2_F_2_ unit is a rather weak Lewis base center, since one may expect significant electron charge shift from this bond to the fluorine substituents. On the other hand, in the C_2_Li_2_H^+^ Lewis acid unit, the proton strongly interacts with the CC electron reach bond (lithium substituents donate electron charge). Hence the interaction with the C_2_F_2_ species results in only the weak polarization of the multi-center bond of the C_2_Li_2_H^+^ unit.

Table 2 shows the medium-strength interactions of lithium complexes with dihydrogen, where the −E_int_ ranges from 3.3 to 5.3 kcal/mol. For the C_2_H_3_^+^-H_2_, C_2_Li_2_H^+^-H_2_ and C_2_F_2_H^+^-H_2_ complexes, weak interactions with −E_int_ between 0.6 and 2.9 kcal/mol are observed. The deformation energies of the complexes of dihydrogen do not exceed 0.1 kcal/mol.

The interaction energy terms are calculated here within the SAPT approach [13]; Table 3 presents the major contributions of the SAPT energy resulting from the grouping of terms of Equation (4) (the terms of Equation (4) are shown in Table S1, Supplementary Information). For the complexes analyzed in this study, the SAPT total interaction energy, $E_{int}^{SAPT2}$, correlates well with the MP2 interaction energy, E_int_ (Table 2), since the linear correlation coefficient, R^2^, is equal to 0.997. This guarantees that the SAPT results are consistent with others presented in this study.

Table 3 shows that the induction energy, *E*_ind_, is the most important attractive interaction energy term for the majority of complexes. In a few cases, the electrostatic energy, *E*_elst_, is the most important attractive term, as in the following complexes: C_2_H_2_Li^+^-C_2_H_2_, C_2_Li_3_^+^-C_2_H_2_ and C_2_Li_3_^+^-C_2_Li_2_. In two former complexes, the *E*_ind_ and *E*_elst_ terms are comparable to each other, at least roughly. However, in the C_2_Li_3_^+^-C_2_Li_2_ complex, the |*E*_elst_| value is more than three times greater than the |*E*_ind_| value. Only in two complexes, C_2_Li_2_H^+^-C_2_F_2_ and C_2_Li_2_H^+^-H_2_, is the dispersion energy the most important attractive contribution. However, very weak interactions link these complexes, since $E_{int}^{SAPT2}$ is equal to −1.5 kcal/mol and −0.6 kcal/mol, respectively.

**Figure 2:**
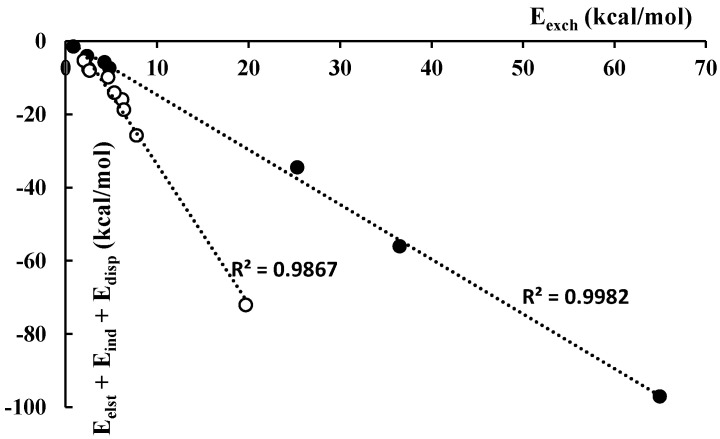
The relationships between the exchange energy term and the sum of attractive energy terms (both in kcal/mol); black circles correspond to the proton-bound complexes and white circles to the lithium cation-bound ones; for both sub-samples the linear correlations are presented.

The importance of the induction interaction for the majority of complexes analyzed here indicates that they are partly covalent in nature. It has been discussed before that the covalent character of hydrogen bonds is connected with interaction energy terms related to electron charge transfers [2]. The increase in importance of such attractive energy terms is a consequence of the increase in exchange repulsion [4]. It is worth mentioning, however, that the increase in the exchange energy results in the increase in all attractive energy terms [4]. This is supported by Figure 2, which presents the linear correlations between the exchange energy and the sum of all attractive energy terms for the proton-bound and the lithium-bound species.

The role of the interactions related to electron charge shifts, which was mentioned above here, was discussed in other former studies. For example, it was pointed out that the occurrence of σ-holes which are crucial in numerous types of intra- and intermolecular interactions is a consequence of the electron charge shifts [38,39,40]. The latter ones are related to polarization interaction energies (the induction interaction energy in the SAPT approach applied in this study).

### 3.3. QTAIM Parameters

The QTAIM approach [11,12] was applied to characterize the π-H∙∙∙π/σ and π-Li∙∙∙π/σ interactions. Figure 1 shows molecular graphs of selected complexes analyzed here. The hydrogen or lithium central attractor is connected in these complexes by bond paths with two critical points of two neighbouring species. Various kinds of such connections occur. For example, for the C_2_H_3_^+^-C_2_H_2_ complex (Figure 1a), the H-center attractor is connected with two non-nuclear attractors (NNAs) located at the CC bond paths of acetylene molecules. One can see that for each acetylene molecule of this complex, two bond critical points (BCPs) occur between C-attractors, and additionally the NNA is located between these BCPs. A similar situation is observed for other complexes, for example, for the C_2_H_2_Li^+^-C_2_H_2_ complex (Figure 1c), where the Li-attractor is connected by bond paths with two NNAs. However, other connections are also observed. For example, only one BCP is located between H-attractors in the dihydrogen molecule. For the C_2_H_2_Li^+^-H_2_ complex, the Li-attractor is linked with the BCP of dihydrogen and with the NNA of acetylene (Figure 1g). In the C_2_H_3_^+^-H_2_ complex (Figure 1e), the central H-attractor is linked with two BCPs, of acetylene and of dihydrogen. It is worth noting that the occurrence of the BCP or the NNA surrounded by two BCPs in the CC acetylene bond path seems to be dependent on the level of calculations [41].

There are other links, especially for the C_2_F_2_H^+^ unit interacting with the π-electron or σ-electron system. In the C_2_F_2_H^+^-C_2_F_2_ complex (Figure 1b), the H-center is situated between carbon atoms. For the C_2_F_2_H^+^-H_2_ complex (Figure 1f), the H-center is connected with the C-atom of the C_2_F_2_ molecular fragment and with the BCP of the dihydrogen. In the case of the C_2_H_2_Li^+^-C_2_F_2_ complex (Figure 1d), the Li-attractor is connected with two NNAs located in the CC bond paths; however, an additional bond path that links hydrogen and fluorine attractors is also detected. Similar molecular graphs are observed for the C_2_F_2_Li^+^-C_2_F_2_ complex, where the additional bond path links the fluorine attractors (Figure S1 in Supplementary Information). The occurrence of the H∙∙∙F bond path for the C_2_H_2_Li^+^-C_2_F_2_ complex seems to be justified, since the centers of the opposite charges are in contact here. However, the F∙∙∙F bond path for the C_2_F_2_Li^+^-C_2_F_2_ complex seems to be controversial, and requires additional analyses. On one hand, it is stated that the bond path occurs for the local stabilizing interactions [42,43,44]; on the other hand, such meaning of this term is questioned [45,46].

Table 4 presents the characteristics of BCPs corresponding to bond paths that connect the hydrogen or lithium attractor with neighbouring molecules. The electron density at BCP, ρ_BCP_, and the total electron energy density at BCP, H_BCP_, are presented there. One of the bond paths of the central attractor corresponding to the H^+^ or Li^+^ cation possesses a BCP characterized by a greater ρ_BCP_ value, thus it corresponds to a stronger interaction. The other bond path contains a BCP where a lower value of ρ_BCP_ is observed, thus this link corresponds to a weaker interaction. However, for a few complexes, two equivalent bond paths linking the H or Li attractor with neighbours are observed, and these are the C_2_F_2_H^+^-C_2_F_2_, C_2_H_2_Li^+^-C_2_H_2_, C_2_F_2_Li^+^-C_2_F_2_ and C_2_Li_3_^+^-C_2_Li_2_ complexes.

It is worth noting that for systems that may be considered as proton-bound complexes, one of the above-mentioned interactions possesses the character of the covalent bond. The corresponding BCP for this interaction is characterized by the negative value of the Laplacian of electron density, ∇^2^ρ_BCP_. This means this interaction may be treated as a multi-center covalent bond since the H-center is linked with a two-center CC bond.

In the C_2_F_2_H^+^-C_2_F_2_ species, the H-center is linked with carbon atoms of two C_2_F_2_ fragments. Both BCPs of the corresponding C∙∙∙H bond paths possess negative ∇^2^ρ_BCP_ values. Hence, according to the QTAIM approach, the H-atom may be considered as the divalent center. The values of the electron density at BCPs corresponding to the above-mentioned C∙∙∙H bond paths are equal to 0.126 au; this is the value typical for BCPs of covalent bonds [11,12]. Note that for this complex, the interaction energy, E_int_, corresponding to the C∙∙∙H short contact is large, equal to −29.4 kcal/mol; the deformation resulting from complexation reduces the latter value significantly. This has been discussed earlier here.

In the case of two complexes, C_2_H_3_^+^-C_2_H_2_ and C_2_H_3_^+^-C_2_F_2_, multi-center covalent bonds are observed as well as weaker interactions, which are partly covalent in nature, and the H_BCP_ values are negative here. For the remaining proton-bound complexes, the C_2_Li_2_H^+^-C_2_F_2_ complex, and complexes of dihydrogen, the H_BCP_ values are positive for the weaker interaction. The latter results indicating weak interactions are in line with the |E_int_| values (Table 2), which do not exceed 3 kcal/mol.

For all lithium cation complexes, the ∇^2^ρ_BCP_ and H_BCP_ values are positive for BCPs corresponding to links between the Li-center and neighbours in spite of some interactions being rather strong (Table 2 and Table 3). Hence, one may expect that for the lithium complexes, the interactions of the Li^+^ cation with neighbouring systems are electrostatic in nature. However the SAPT results discussed earlier here do not support that, because induction and dispersion contributions also play a significant role for these complexes.

The QTAIM charges of complexes analyzed are presented in Table 5. There is a significant difference between the proton-bound complexes and the lithium systems. For the former complexes, the proton located in the center withdraws electron charge from neighbouring species, and its charge amounts to 0.217–0.395 au. In the case of lithium species, the Li-charge amounts to 0.900–0.954 au, which is close to unity.

Let us analyze the C_2_H_3_^+^-C_2_H_2_ complex more closely. It is composed of a C_2_H_3_^+^ cation and a C_2_H_2_ acetylene molecule; if these units are isolated, they possess charges +1 au and 0, respectively. In the complex, the electron charge of −0.704 au (Table 5) is transferred from the Lewis base (C_2_H_2_) into the C_2_H_3_^+^ cation. One can see that meaningful electron charge transfer from the Lewis base unit into the Lewis acid takes place for some complexes—for the proton-bound complexes and for the lithium systems. Very small transfer is observed for complexes of dihydrogen. However, even for the latter complexes the induction interaction energy, *E*_ind_^(2)^, which is related to the electron charge shifts, makes an important contribution to the total interaction energy. This may be explained in the following way. Electron charge shifts resulting from complexation are not only connected with transfers from neutral dihydrogen into the cation (Li^+^ or H^+^ connected with the π-electron system), but also with transfers within interacting species.

Let us look at the charges of the separated C_2_H_3_^+^ and C_2_H_2_Li^+^ units; both possess a charge equal to +1 au. For the former species, the charge distribution is as follows: +0.688 au (C_2_H_2_ fragment) and +0.312 au (H^+^ formal proton). In the case of the C_2_H_2_Li^+^ unit, the following distribution is observed: +0.037 au (C_2_H_2_ fragment) and +0.963 au (Li^+^ formal cation). Therefore, for the C_2_H_3_^+^ cation, large electron charge transfer from the acetylene molecule to the proton occurs; this is not “a pure proton” anymore. In the case of the C_2_H_2_Li^+^ species, only a slight electron charge shift to the lithium cation is observed. Thus, the differences in the charges of the H and Li centers of complexes discussed here (Table 5) are connected with primary cations of +1 au charge, which further additionally interact with the Lewis base units.

**Figure 3:**
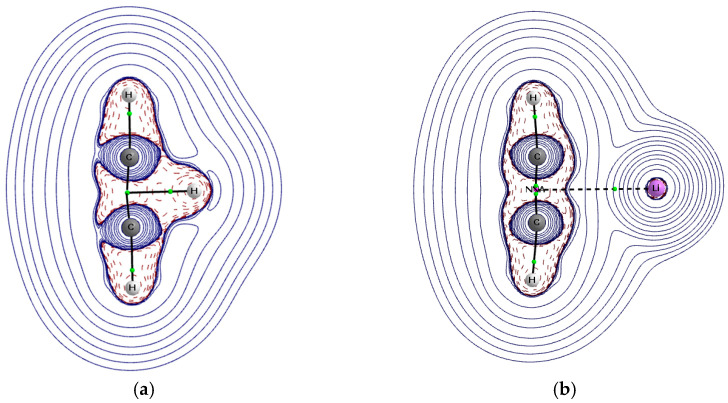
The contours of the Laplacian of electron density, ∇^2^ρ, for the (**a**) C_2_H_3_^+^ cation and (**b**) C_2_H_2_Li^+^ cation. Continuous lines correspond to the positive values of ∇^2^ρ, while broken lines correspond to negative values.

Figure 3 shows the Laplacian of electron density isolines for the C_2_H_3_^+^ and C_2_H_2_Li^+^ cations. For the former cation, the H-center is connected with the acetylene molecule by the bond path that lies within the region of negative values of the Laplacian of electron density. The H-BCP bond path mimics the two-center covalent bond; its formation requires enormous electron charge transfer. In the C_2_H_2_Li^+^ cation, the Li-center is connected with the acetylene by the Li-NNA bond path that is almost completely located in the region of the positive values of Laplacian, including the corresponding BCP. This means the interaction related to this bond path does not possess covalent characteristics, but rather ionic. It is also related to the slight electron charge transfer between the Li^+^ and C_2_H_2_.

Let us analyze the C_2_H_3_^+^-C_2_H_2_ complex where the connection of C_2_H_3_^+^ and C_2_H_2_ units leads to the electron charge transfer of −0.704 au from the neutral C_2_H_2_ molecule into the C_2_H_3_^+^ cation (Table 5). As a consequence, the C_2_H_3_^+^ fragment in the complex possesses the charge of +0.296 au. The charge of H-center increases, from +0.312 au in the isolated C_2_H_3_^+^ to +0.395 au in the C_2_H_3_^+^-C_2_H_2_ complex, while the charge of the acetylene fragment of the C_2_H_3_^+^ cation in the complex amounts to −0.099 au. This is a similar situation to that observed for the typical A-H∙∙∙B hydrogen bonds, where, in the isolated unit, the H-center is positively charged, and in the complex this charge increases in spite of the electron charge transfer from the B-unit to the A-H one. However the main part of this electron charge is shifted to the A-fragment. This is connected with the rehybridization process described in several studies [3,47,48].

It is worth noting that for the A-H∙∙∙B hydrogen bond, complexation usually leads to the elongation of the A-H bond that is connected with the shift of the corresponding A-H stretch band to a lower frequency (red shift) [3,49]. However, the less common shortening of the A-H bond resulting from the formation of the hydrogen bond also occurs, and this is connected with the shift of the corresponding A-H stretch band to a higher frequency (blue shift) [49,50,51,52,53,54]. In the case of the C_2_H_3_^+^-C_2_H_2_ complex, the H∙∙∙C distances are equal to 1.405 and 1.406 Å for the acetylene molecule situated closer to H-center than the other acetylene (Table 1). Hence the elongation of these distances is observed, since they are equal to 1.276 Å in the isolated C_2_H_3_^+^ cation. One can see that the π-H∙∙∙π link possesses numerous characteristics of the hydrogen bond in the C_2_H_3_^+^-C_2_H_2_ complex, similarly to the π-H∙∙∙π and π-H∙∙∙σ interactions in other complexes discussed in this study.

### 3.4. NCI Approach Results

The NCI approach is based on the topology of electron density, ρ, and it enables the identification and analysis of various intra- and intermolecular interactions [14,15,29]. One of the useful results from the NCI method applies to the 2D graph of the reduced density gradient (RDG) against the sign(λ_2_)ρ product. The λ_2_ parameter is the second eigenvalue of the Hessian matrix of the ρ density. This approach is usually applied for low ρ densities, and is characteristic of weak interactions. There are three kinds of spikes in the RDG plot. (1) Those of large negative values of sign(λ_2_)ρ (ρ > 0, λ_2_ < 0), which correspond to attractive interactions such as hydrogen bonds and other Lewis acid–Lewis base interactions. (2) The spikes at large positive values of sign(λ_2_)ρ (ρ > 0, λ_2_ > 0), which indicate repulsive interactions, as for example those occurring within the rings, or those between the centers of similar charge. (3) The spikes at sign(λ_2_)ρ values tending to zero occur for very weak interactions, usually van der Waals ones (vdWs), driven mainly by dispersion forces. One can see that taking into account the sign(λ_2_)ρ value instead of the electron density, ρ, allows one to distinguish between repulsive and attractive interactions, which are characterized by positive and negative sign(λ_2_) values, respectively.

**Figure 4:**
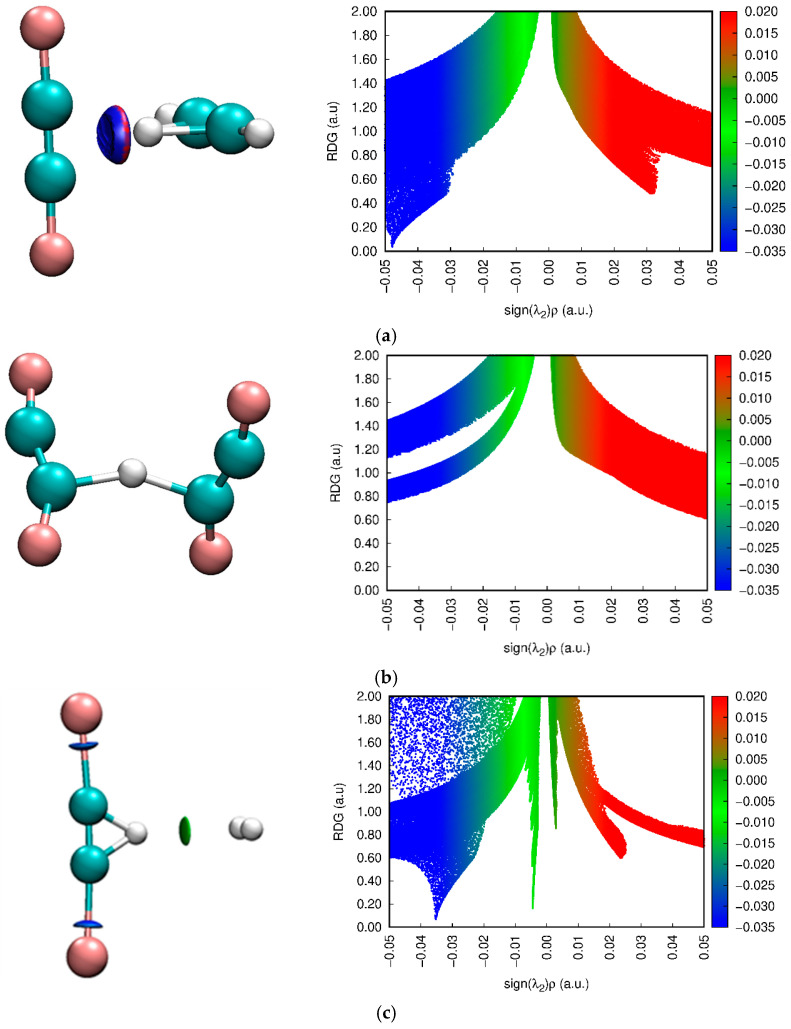
The RDG plots (**right**) and the corresponding molecular systems (**left**) of selected proton-bound complexes. The following complexes are presented: (**a**) C_2_H_3_^+^-C_2_F_2_, (**b**) C_2_F_2_H^+^-C_2_F_2_, (**c**) C_2_Li_2_H^+^-H_2_.

**Figure 5:**
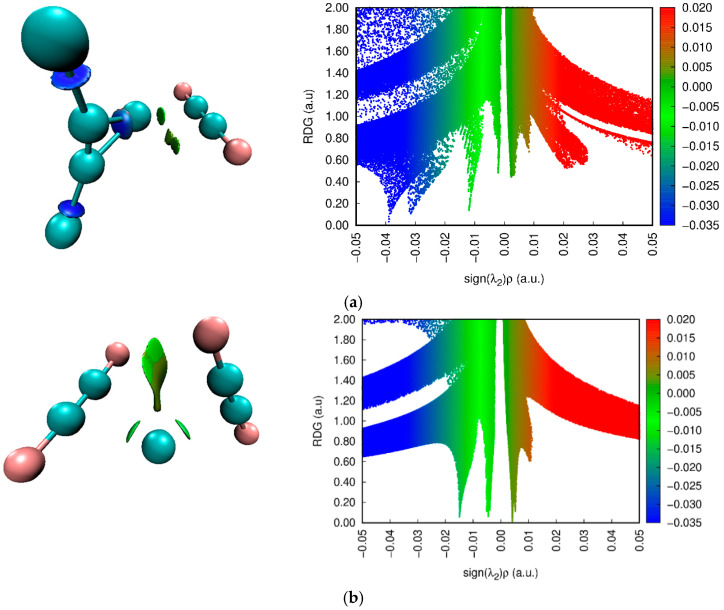

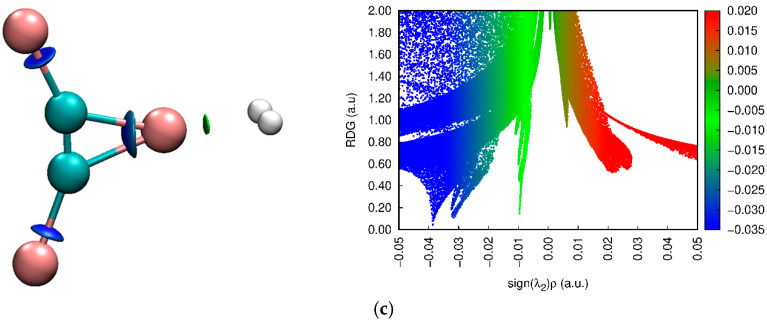
The RDG plots (**right**) and the corresponding molecular systems (**left**) of selected lithium-bound complexes. The following complexes are presented: (**a**) C_2_Li_3_^+^-C_2_F_2_, (**b**) C_2_F_2_Li^+^-C_2_F_2_, (**c**) C_2_Li_3_^+^-H_2_.

Figure 4 and Figure 5 show the RDG plots and the corresponding molecular structures, where various types of interactions are revealed. The range of sign(λ_2_)ρ value between −0.05 and +0.05 a.u. for the RDG plots, and the RDG value of 0.5 representing the surfaces in figures of molecular structures, were chosen to indicate various so-called noncovalent interactions. The RDG plots for all complexes analyzed here are presented in Figure S2 in the Supplementary Information. The selected complexes with broader sign(λ_2_)ρ ranges are presented in Figure S3. In all these figures, attractive, repulsive, and weak interactions (mostly classified as vdWs) are shown in blue, red and green colors, respectively. Figure 4 presents cases of proton-bound complexes. The C_2_H_3_^+^-C_2_F_2_ complex is shown in Figure 4a. One can see here the RDG spike at sign(λ_2_)ρ value close to −0.05 au. It corresponds to the H∙∙∙π interaction, which is indicated by the disc in the molecular structure presented in this figure. This is the interaction between the H-center and the π-electron system of the C_2_F_2_ molecule. The disc shape presented in Figure 4a also refers to the influence of repulsive interaction. The latter is reflected in the RDG plot, since the spike occurs at a sign(λ_2_)ρ value close to +0.03 au. One can note that another H∙∙∙π interaction is not indicated in this figure, since it corresponds to a much stronger multi-center covalent π-H interaction, which is outside the range applied here. A very similar picture to the C_2_H_3_^+^-C_2_F_2_ complex is observed for the C_2_H_3_^+^-C_2_H_2_ complex (Figure S2).

Figure 4b shows the C_2_F_2_H^+^-C_2_F_2_ complex; the weak interactions are not detected here. Only covalent bonds outside the sign(λ_2_)ρ range applied occur. This complex may be considered as the C_4_F_4_H^+^ cation, where even the hydrogen center is divalent. As was discussed earlier here, both short H∙∙∙C contacts are characterized by negative ∇^2^ρ_BCP_ values. The C_2_Li_2_H^+^-H_2_ complex (Figure 4c) is another interesting system. The spike of RDG occurs for sign(λ_2_)ρ values close to −0.005 au, and this corresponds to the H∙∙∙σ interaction between the H-center and the dihydrogen—this is observed in the complex structure. Stronger carbon–lithium interactions are also observed for this structure, and a corresponding spike occurs in the RDG plot for sign(λ_2_)ρ values close to −0.04 au. These C∙∙∙Li interactions that are stronger than the H∙∙∙σ interaction correspond to the strongly polarized C-Li bonds. Such a situation is observed for other complexes discussed in this study, where the C_2_Li_2_ molecular fragment occurs. This occurs in the C_2_Li_3_^+^-C_2_F_2_ and C_2_Li_3_^+^-H_2_ complexes discussed further here (see Figure 5a,c, respectively), as well as in the C_2_Li_2_H^+^-C_2_F_2_ complex (see Figure S2 in Supplementary Information).

Figure 5 presents lithium cation complexes. The C_2_Li_3_^+^-C_2_F_2_ complex is shown in Figure 5a. One can see here two C∙∙∙Li interactions and one Li∙∙∙π interaction, which are marked by their molecular structure as stronger ones (blue color). The RDG spikes occur here for sign(λ_2_)ρ values close to −0.04 au and −0.03 au, respectively. The latter Li∙∙∙π interaction may be treated as the more strongly polarized multi-center bond (in another way it may be marked as Li∙∙∙CC or τ_CLiC_). Three weak interactions are also marked in the molecular structure between the central Li-cation and the C_2_F_2_ molecule, and a spike in sign(λ_2_)ρ value at −0.01 au and another closer to zero, but still negative, correspond to these interactions in the RDG plot. The former spike is related to the Li∙∙∙π (π-electrons of C_2_F_2_) contact and the second one corresponds to very weak Li∙∙∙F interactions. These interactions are accompanied by repulsive components.

The C_2_F_2_Li^+^-C_2_F_2_ complex (Figure 5b) was discussed in the previous section in terms of the QTAIM approach. For this system, a F∙∙∙F bond path with a corresponding bond critical point is observed. The NCI method shows here a very weak attractive interaction, since the spike of RDG at about −0.005 au sign(λ_2_)ρ value is observed. This is reflected by the leaf-shaped surface that occurs between fluorine centers. This interaction is accompanied by repulsion forces (the spike at +0.005 au on the 2D RDG plot). One may expect that the local F∙∙∙F interaction is mainly governed by dispersion and repulsion forces. A very similar situation occurs for the C_2_H_2_Li^+^-C_2_F_2_ complex (see its molecular graph in Figure 1d). However, here, an attractive H∙∙∙F interaction occurs, where the atoms in contact possess opposite charges. Thus, the latter local interaction is mainly steered by electrostatic forces. For the C_2_F_2_Li^+^-C_2_F_2_ complex, two Li∙∙∙π interactions stronger than F∙∙∙F but still weak occur, with the corresponding spike at −0.015 au sign(λ_2_)ρ value (surfaces of the molecular structure are also observed).

Figure 5c shows interactions in the C_2_Li_3_^+^-H_2_ complex. The molecular structure is accompanied by three surfaces that correspond to medium-strength interactions; these are two Li∙∙∙C interactions, which are reflected by the RDG spike at around −0.04 au in the value of sign(λ_2_)ρ. One additional surface corresponds to the multi-center Li∙∙∙π interaction—the corresponding spike appears here at about −0.03 au. One may expect that these interactions are electrostatic in nature, occurring between cationic forms of lithium and the anionic C_2_ molecular fragment. For this C_2_Li_3_^+^-H_2_ complex, the QTAIM charges for Li-centers attached to carbons are equal to +0.939 au (each one possesses such a charge); the Li-center interacting with π-electrons of the C_2_ fragment has a charge amounting to +0.923 au, and this C_2_ fragment possesses a charge equal to −1.809 au. In other words, one may assume that these are the interactions of three Li^+^ cations with the C_2_^2−^ anion. The whole C_2_Li_3_^+^ unit has +0.992 au QTAIM charge in the C_2_Li_3_^+^-H_2_ complex. The molecular hydrogen, H_2_, that acts in this complex as the Lewis base unit loses its electron charge, and becomes slightly positively charged (+0.008 au). The dihydrogen interaction with Li-center, Li∙∙∙σ, is very weak; the corresponding surface shown in Figure 5c occurs, and a spike emerges in the RDG plot at the −0.01 au sign(λ_2_)ρ value.

## 4. Conclusions

Two groups of species were analyzed here: the proton-bound complexes connected by the π-H∙∙∙π and π-H∙∙∙σ links, and their counterparts with a lithium cation instead of an H-center. The connections with the H-center possess numerous characteristics of hydrogen bonds. They may be classified as hydrogen bonds, as in line with the definition introduced recently. This states that “the hydrogen bond is a local stabilizing interaction between the proton or the electron charge deficient region of hydrogen and the electron rich region attributed to one or more centers.” [49]. In such a way, the proton inserted between two π-electron systems participates in two H∙∙∙π hydrogen bonds, while the proton located between π-electron and σ-electron systems participates in one H∙∙∙π hydrogen bond and one H∙∙∙σ hydrogen bond.

On the other hand, for all proton-bound complexes, one interaction is usually stronger than the other. For the sample of complexes analyzed here, the stronger interaction is of the π-H type. It possesses numerous characteristics of the hydrogen bond, but it may also be classified as a multi-center covalent bond. The energetic parameters, as well as the SAPT, QTAIM and NCI approaches, confirm the above statements.

Quite a different situation is observed for the lithium-bound complexes, where this cation possesses positive charge close to unity. The Li∙∙∙π and Li∙∙∙σ interactions in these complexes do not possess the characteristics of covalent bonds. The π-Li∙∙∙π and π-Li∙∙∙σ connections may be considered lithium bonds [55,56,57]. However, they possess different characteristics to their hydrogen counterparts.

## Figures and Tables

**Table 1:** Distances between the proton or lithium center and neighbouring carbon atoms for π-electron species and neighbouring hydrogen atoms for dihydrogen complexes (in Å); see Scheme 1, where distances are defined. If the cation is inserted between π-electron systems, R_1_ and R_2_ distances usually correspond to shorter contacts. For the dihydrogen complexes, R_3_ and R_4_ correspond to H/Li∙∙∙H distances.

Complex	R_1_	R_2_	R_3_	R_4_
Protonated systems				
C_2_H_3_^+^	1.276	1.276	-	-
C_2_F_2_H^+^	1.095	2.004	-	-
C_2_Li_2_H^+^	1.254	1.254	-	-
C_2_H_3_^+^-C_2_H_2_	1.405	1.406	1.828	1.828
C_2_H_3_^+^-C_2_F_2_	1.363	1.364	1.927	1.929
C_2_F_2_H^+^-C_2_F_2_	1.394	1.916	1.394	1.916
C_2_Li_2_H^+^-C_2_F_2_	1.259	1.259	2.593	2.595
C_2_H_3_^+^-H_2_	1.286	1.286	1.942	1.942
C_2_F_2_H^+^-H_2_	1.100	1.995	2.117	2.117
C_2_Li_2_H^+^-H_2_	1.255	1.255	2.569	2.569
Lithium species				
C_2_H_2_Li^+^	2.327	2.327	-	-
C_2_F_2_Li^+^	2.438	2.438	-	-
C_2_Li_3_^+^	2.082	2.082	-	-
C_2_H_2_Li^+^-C_2_H_2_	2.342	2.342	2.342	2.342
C_2_H_2_Li^+^-C_2_F_2_	2.323	2.336	2.442	2.472
C_2_F_2_Li^+^-C_2_F_2_	2.436	2.447	2.436	2.448
C_2_Li_3_^+^-C_2_H_2_	2.102	2.102	2.397	2.397
C_2_Li_3_^+^-C_2_F_2_	2.091	2.091	2.496	2.508
C_2_Li_3_^+^-C_2_Li_2_	2.174	2.175	2.175	2.175
C_2_H_2_Li^+^-H_2_	2.332	2.333	2.068	2.071
C_2_F_2_Li^+^-H_2_	2.444	2.445	2.059	2.062
C_2_Li_3_^+^-H_2_	2.089	2.089	2.126	2.126

**Table 2:** The interaction, binding and deformation energies, E_int_, E_bin_ (corrected for BSSE) and E_def_, respectively, for complexes analyzed in this study. The BSSE correction is included (all in kcal/mol).

Complex	E_int_	E_bin_	E_def_	BSSE
Protonated systems				
C_2_H_3_^+^-C_2_H_2_	−20.3	−15.9	4.3	1.1
C_2_H_3_^+^-C_2_F_2_	−10.7	−8.3	2.4	1.2
C_2_F_2_H^+^-C_2_F_2_	−29.4	−5.6	23.9	1.9
C_2_Li_2_H^+^-C_2_F_2_	−1.5	−1.5	0.0	0.7
C_2_H_3_^+^-H_2_	−2.9	−2.9	0.1	0.2
C_2_F_2_H^+^-H_2_	−1.8	−1.8	0.0	0.2
C_2_Li_2_H^+^-H_2_	−0.6	−0.6	0.0	0.1
Lithium species				
C_2_H_2_Li^+^-C_2_H_2_	−17.9	−17.6	0.3	0.6
C_2_H_2_Li^+^-C_2_F_2_	−8.7	−8.4	0.3	0.9
C_2_F_2_Li^+^-C_2_F_2_	−9.6	−9.2	0.3	1.0
C_2_Li_3_^+^-C_2_H_2_	−12.2	−12.0	0.2	0.7
C_2_Li_3_^+^-C_2_F_2_	−5.1	−4.9	0.2	0.9
C_2_Li_3_^+^-C_2_Li_2_	−52.0	−49.6	2.4	1.1
C_2_H_2_Li^+^-H_2_	−5.0	−5.0	0.0	0.2
C_2_F_2_Li^+^-H_2_	−5.3	−5.3	0.0	0.2
C_2_Li_3_^+^-H_2_	−3.3	−3.3	0.0	0.2

**Table 3:** The interaction energy contributions (in kcal/mol) that are defined by Equation (5) and the total interaction energy, $E_{int}^{SAPT2}$.

Complex	*E*_elst_	*E*_exch_	*E*_ind_	*E*_disp_	$E_{int}^{SAPT2}\;$
Protonated systems					
C_2_H_3_^+^-C_2_H_2_	−19.29	36.50	−27.45	−9.33	−19.56
C_2_H_3_^+^-C_2_F_2_	−7.93	25.30	−19.19	−7.38	−9.19
C_2_F_2_H^+^-C_2_F_2_	−15.12	64.96	−66.38	−15.56	−32.10
C_2_Li_2_H^+^-C_2_F_2_	−1.52	4.24	−1.34	−2.86	−1.48
C_2_H_3_^+^-H_2_	−2.21	4.78	−3.51	−1.57	−2.51
C_2_F_2_H^+^-H_2_	−1.33	2.35	−1.86	−0.88	−1.72
C_2_Li_2_H^+^-H_2_	−0.53	0.84	−0.28	−0.61	−0.58
Lithium species					
C_2_H_2_Li^+^-C_2_H_2_	−13.08	7.75	−11.78	−0.92	−18.03
C_2_H_2_Li^+^-C_2_F_2_	−2.44	5.31	−10.05	−1.61	−8.78
C_2_F_2_Li^+^-C_2_F_2_	−2.66	6.17	−11.04	−2.22	−9.73
C_2_Li_3_^+^-C_2_H_2_	−10.28	6.37	−7.08	−1.36	−12.36
C_2_Li_3_^+^-C_2_F_2_	−2.43	4.63	−5.78	−1.66	−5.24
C_2_Li_3_^+^-C_2_Li_2_	−53.69	19.70	−15.60	−2.82	−52.41
C_2_H_2_Li^+^-H_2_	−2.35	2.51	−4.94	−0.28	−5.07
C_2_F_2_Li^+^-H_2_	−2.42	2.61	−5.33	−0.26	−5.41
C_2_Li_3_^+^-H_2_	−1.94	1.98	−2.97	−0.40	−3.32

**Table 4:** The selected QTAIM parameters (in au), the electron density at BCP, ρ_BCP_, and the total electron energy density at BCP, H_BCP_, are given; the bold values correspond to interactions characterized by negative ∇^2^ρ_BCP_ values. One asterisk (^*^) corresponds to stronger interactions, two asterisks (^**^) correspond to weaker interactions.

Complex	ρ_BCP_ ^*^	H_BCP_ ^*^	ρ_BCP_ ^**^	H_BCP_ ^**^
Protonated systems				
C_2_H_3_^+^-C_2_H_2_	**0.152**	**−0.106**	0.057	−0.020
C_2_H_3_^+^-C_2_F_2_	**0.168**	**−0.120**	0.048	−0.014
C_2_F_2_H^+^-C_2_F_2_	**0.126**	**−0.083**	**0.126**	**−0.083**
C_2_Li_2_H^+^-C_2_F_2_	**0.297**	**−0.159**	0.011	0.001
C_2_H_3_^+^-H_2_	**0.201**	**−0.154**	0.017	0.000
C_2_F_2_H^+^-H_2_	**0.281**	**−0.327**	0.011	0.001
C_2_Li_2_H^+^-H_2_	**0.208**	**−0.161**	0.004	0.001
Lithium species				
C_2_H_2_Li^+^-C_2_H_2_	0.018	0.003	0.018	0.003
C_2_H_2_Li^+^-C_2_F_2_	0.019	0.004	0.013	0.003
C_2_F_2_Li^+^-C_2_F_2_	0.015	0.003	0.015	0.003
C_2_Li_3_^+^-C_2_H_2_	0.031	0.003	0.015	0.003
C_2_Li_3_^+^-C_2_F_2_	0.032	0.003	0.012	0.003
C_2_Li_3_^+^-C_2_Li_2_	0.026	0.003	0.026	0.003
C_2_H_2_Li^+^-H_2_	0.019	0.003	0.012	0.003
C_2_F_2_Li^+^-H_2_	0.015	0.003	0.012	0.003
C_2_Li_3_^+^-H_2_	0.032	0.003	0.010	0.003

**Table 5:** The parameters related to QTAIM charges (in au). The total Lewis acid and Lewis base units’ charges are given (marked as L.acid and L.base, respectively). The charge of the H-atom or Li-atom located in the center of the complex, Q_Li/H_, the part of the Lewis acid unit (without the H or Li center), Q_La-part_.

Complex	L.acid	Q_Li/H_	Q_La-part_	L.base
Protonated systems				
C_2_H_3_^+^-C_2_H_2_	0.296	0.395	−0.099	0.704
C_2_H_3_^+^-C_2_F_2_	0.112	0.366	−0.254	0.888
C_2_F_2_H^+^-C_2_F_2_	0.655	0.311	0.345	0.345
C_2_Li_2_H^+^-C_2_F_2_	0.164	0.222	−0.058	0.836
C_2_H_3_^+^-H_2_	0.978	0.333	0.645	0.022
C_2_F_2_H^+^-H_2_	0.987	0.295	0.692	0.013
C_2_Li_2_H^+^-H_2_	0.999	0.217	0.782	0.001
Lithium species				
C_2_H_2_Li^+^-C_2_H_2_	0.409	0.931	−0.522	0.591
C_2_H_2_Li^+^-C_2_F_2_	0.220	0.938	−0.718	0.780
C_2_F_2_Li^+^-C_2_F_2_	0.972	0.944	0.028	0.028
C_2_Li_3_^+^-C_2_H_2_	0.387	0.911	−0.524	0.613
C_2_Li_3_^+^-C_2_F_2_	0.208	0.916	−0.708	0.792
C_2_Li_3_^+^-C_2_Li_2_	0.950	0.900	0.050	0.050
C_2_H_2_Li^+^-H_2_	0.985	0.948	0.037	0.015
C_2_F_2_Li^+^-H_2_	0.984	0.954	0.030	0.016
C_2_Li_3_^+^-H_2_	0.999	0.923	0.076	0.001

## Data Availability

New results are presented in this article, other data supporting reported in the main article results can be found in the corresponding Supplementary Information.

## Supplementary Materials

The following are available online, Figure S1: Molecular graphs of selected complexes analyzed in this study; big circles correspond to attractors, small green circles to bond critical points, small red circles to ring critical points and to non-nuclear attractors (NNAs). The continuous and broken lines correspond to bond paths. Figure S2: The RDG plots (right) and the corresponding molecular systems (left) of selected proton bound complexes. The following complexes are presented: (a) C_2_H_3_^+^-C_2_F_2_, (b) C_2_F_2_H^+^-C_2_F_2_, (c) C_2_Li_2_H^+^-H_2_, (d) C_2_Li_3_^+^-C_2_F_2_, (e) C_2_F_2_Li^+^-C_2_F_2_, (f) C_2_Li_3_^+^-H_2_; and additional complexes not presented in the main article: (g) C_2_H_3_^+^-C_2_H_2_, (h) C_2_Li_2_H^+^-C_2_F_2_ (i) C_2_H_2_Li^+^-C_2_F_2_, (j) C_2_H_2_Li^+^-C_2_H_2_, (k) C_2_Li_3_^+^-C_2_H_2_, (l) C_2_Li_3_^+^-C_2_Li_2_, (m) C_2_H_3_^+^-H_2_, (n) C_2_F_2_H^+^-H_2_, (o) C_2_H_2_Li^+^-H_2_, (p) C_2_F_2_Li^+^-H_2_. Complexes from (a) to (f) are presented in the main article. The sign(λ_2_)ρ values between −0.05 and +0.05 a.u. The RDG value for surfaces presented at molecular structures is equal to 0.5. Figure S3: The RDG plots (right) and the corresponding molecular systems (left) of selected proton bound complexes. The following complexes are presented: (a) C_2_H_3_^+^-C_2_F_2_, (b) C_2_F_2_H^+^-C_2_F_2_, (c) C_2_Li_2_H^+^-H_2_, (d) C_2_Li_3_^+^-C_2_F_2_, (e) C_2_F_2_Li^+^-C_2_F_2_, (f) C_2_Li_3_^+^-H_2_. The sign(λ_2_)ρ values between −0.1 and +0.1 a.u. The RDG value for surfaces presented at molecular structures is equal to 0.55. Table S1: The interaction energy terms (in kcal/mol) that are defined by equation 4 (main article); $E_{int}^{HF}$, is the Hartree-Fock interaction energy.

## Sample Availability

Samples of the compounds are not available from the authors.
