# Supplementary material for: Hydrogen and Lithium Bonds—Lewis Acid Units Possessing Multi-Center Covalent Bonds
Source: Molecules. 2021 Nov 17;26(22):6939. doi: 10.3390/molecules26226939 (PMC8624437; doi:10.3390/molecules26226939)
Supplement: Supplementary file 1 [file molecules-26-06939-s001.zip › molecules-1446866-supplementary.pdf]

Supplementary Information

Hydrogen and lithium bonds – Lewis acid units possessing multi-centre covalent bonds

by

Mohammad Aarabi, Samira Gholami and Sławomir J. Grabowski *

Mohammad Aarabi - Dipartimento di Chimica Industriale "Toso Montanari", Università degli Studi di Bologna, Viale del Risorgimento 4, I-40136 Bologna, Italy
orcid.org/0000-0001-8017-3806; e-mail: mohammad.aarabi@unibo.it
Samira Gholami - Dipartimento di Chimica Industriale "Toso Montanari", Università degli Studi di Bologna, Viale del Risorgimento 4, I-40136 Bologna, Italy
orcid.org/0000-0002-8133-0890; e-mail: samira.gholami2@unibo.it
Sławomir J. Grabowski - Faculty of Chemistry, University of the Basque Country and Donostia International Physics Center (DIPC), P.K. 1072, 20080 Donostia, Spain
IKERBASQUE, Basque Foundation for Science, 48011 Bilbao, Spain
orcid.org/0000-0003-4161-2938
e-mail: s.grabowski@ikerbasque.org

Abstract MP2/aug-cc-pVTZ calculations were carried out on complexes where the proton or the lithium cation is located between π-electron systems or it is located between π-electron and σ-electron units. The acetylene and its fluorine or lithium derivatives act as the Lewis base π-electron species similarly as the molecular hydrogen that acts as the electron donor by its σ-electrons. These complexes may be classified as linked by π-H···π/σ hydrogen bonds and π-Li···π/σ lithium bonds. Properties of these interactions are discussed; particularly Lewis acid units are analysed because multi-centre π-H or π-Li covalent bonds may occur in these systems. Various theoretical approaches were applied here to analyse the above mentioned interactions; the Quantum Theory of Atoms in Molecules (QTAIM), the Symmetry-Adapted Perturbation Theory (SAPT) and the Non-Covalent Interaction (NCI) method.

Key words: hydrogen bond, lithium bond, multi-centre covalent bond QTAIM approach, SAPT approach, NCI method

Figure S1 Molecular graphs of selected complexes analysed in this study; big circles correspond to attractors, small green circles to bond critical points, small red circles to ring critical points and to non-nuclear attractors (NNAs). The continuous and broken lines correspond to bond paths.

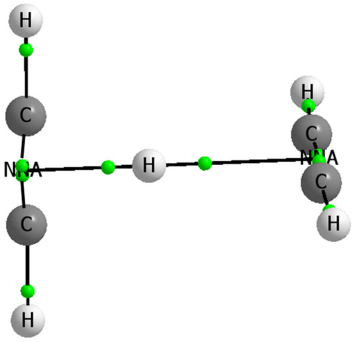

$C_2H_3^+$-C_2H_2

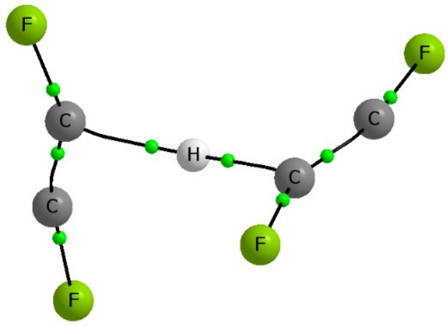

$C_2F_2H^+$-C_2F_2

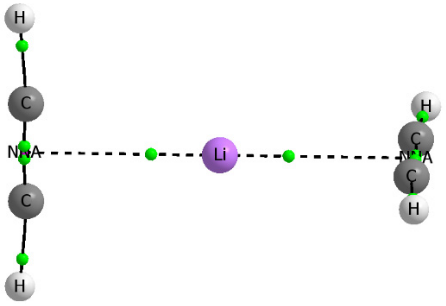

$C_2H_2Li^+$-C_2H_2

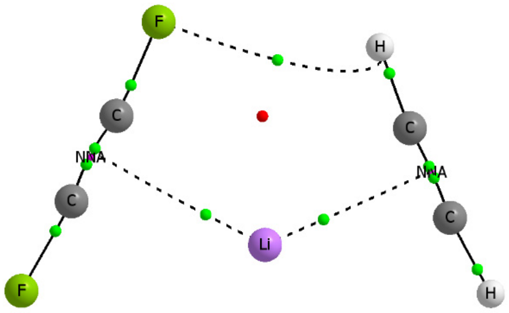

$C_2H_2Li^+$-C_2F_2

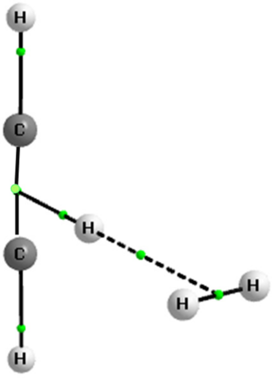

$C_2H_3^+$-H_2

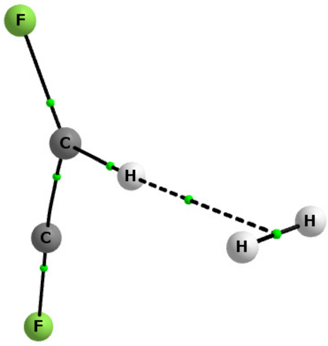

$C_2F_2H^+$-H_2

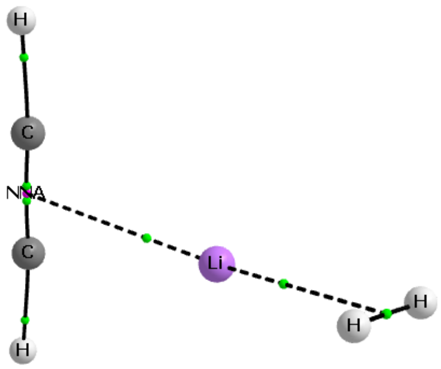

$C_2H_2Li^+$-H_2

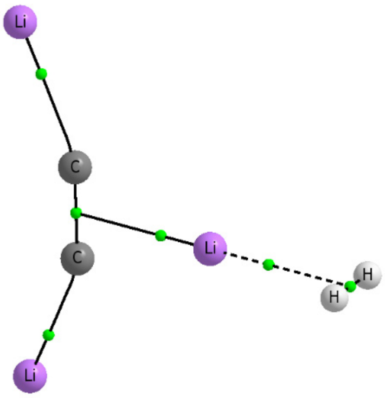

$C_2Li_3^+$-H_2

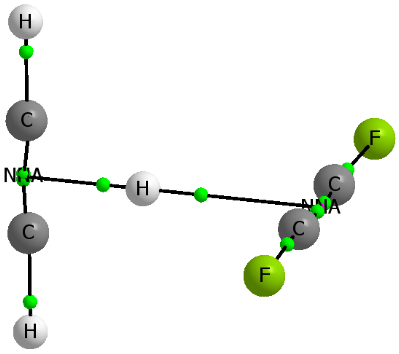

$C_2H_3^+$-C_2F_2

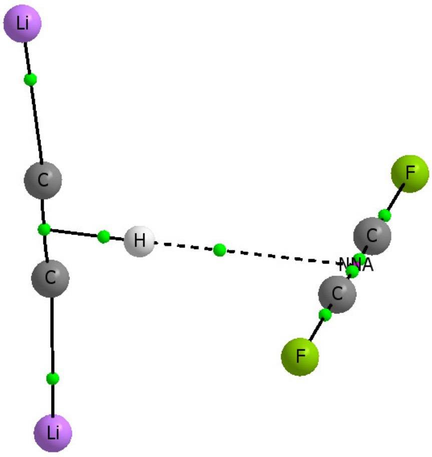

$C_2Li_2H^+$-C_2F_2

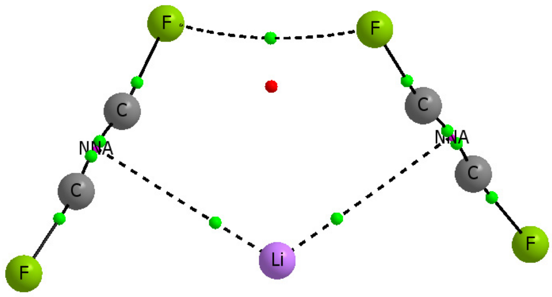

$C_2F_2Li^+$-C_2F_2

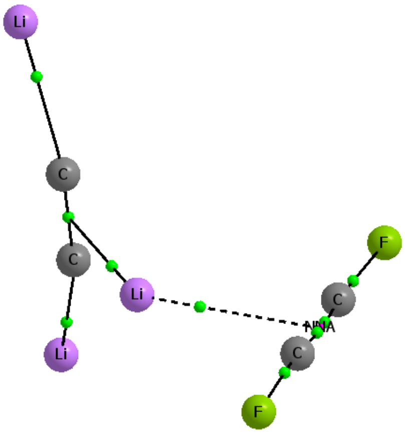

$C_2Li_3^+$-C_2F_2

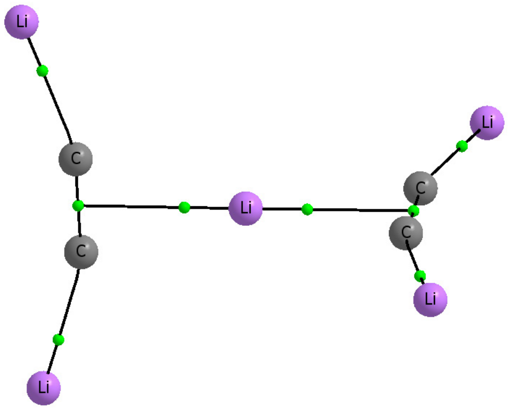

$C_2Li_3^+$-C_2Li_2

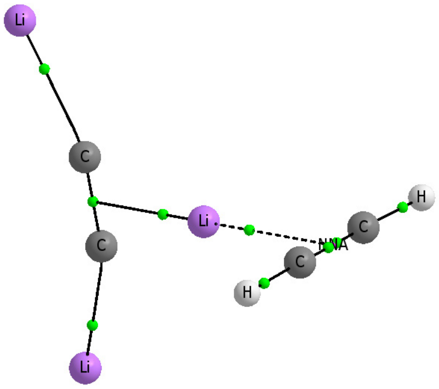

$C_2Li_3^+$-C_2H_2

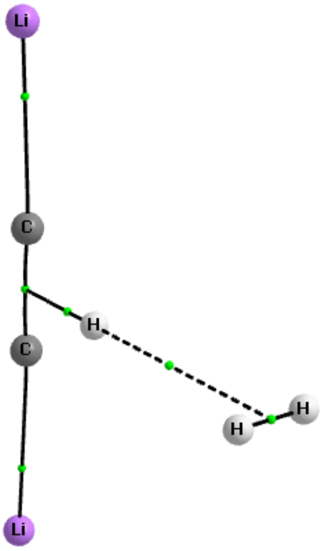

$C_2Li_2H^+$-H_2

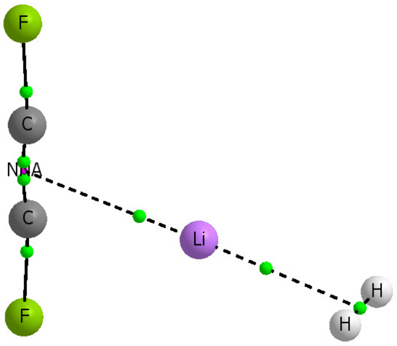

$C_2F_2Li^+$-H_2

Figure S2 The RDG plots (right) and the corresponding molecular systems (left) of selected proton bound complexes. The following complexes are presented: (a) $C_2H_3^+$-C_2F_2, (b) $C_2F_2H^+$-C_2F_2, (c) $C_2Li_2H^+$-H_2, (d) $C_2Li_3^+$-C_2F_2, (e) $C_2F_2Li^+$-C_2F_2, (f) $C_2Li_3^+$-H_2; and additional complexes not presented in the main article: (g) $C_2H_3^+$-C_2H_2, (h) $C_2Li_2H^+$-C_2F_2 (i) $C_2H_2Li^+$-C_2F_2, (j) $C_2H_2Li^+$-C_2H_2, (k) $C_2Li_3^+$-C_2H_2, (l) $C_2Li_3^+$-C_2Li_2, (m) $C_2H_3^+$-H_2, (n) $C_2F_2H^+$-H_2, (o) $C_2H_2Li^+$-H_2, (p) $C_2F_2Li^+$-H_2. Complexes from (a) to (f) are presented in the main article. The sign(λ_2)ρ values between -0.05 and +0.05 a.u. The RDG value for surfaces presented at molecular structures is equal to 0.5.

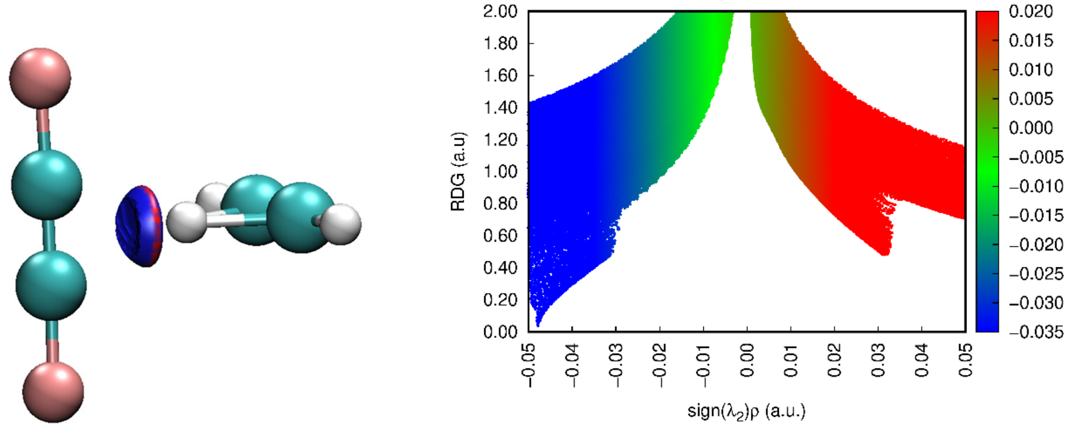

a

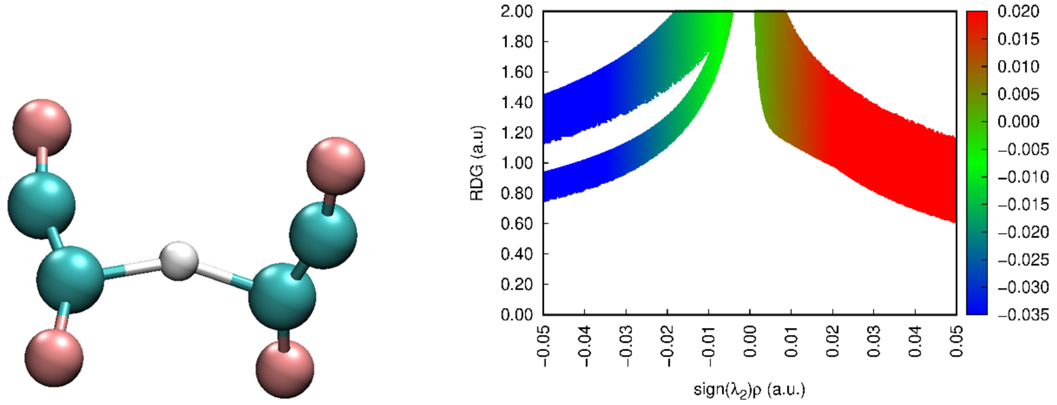

b

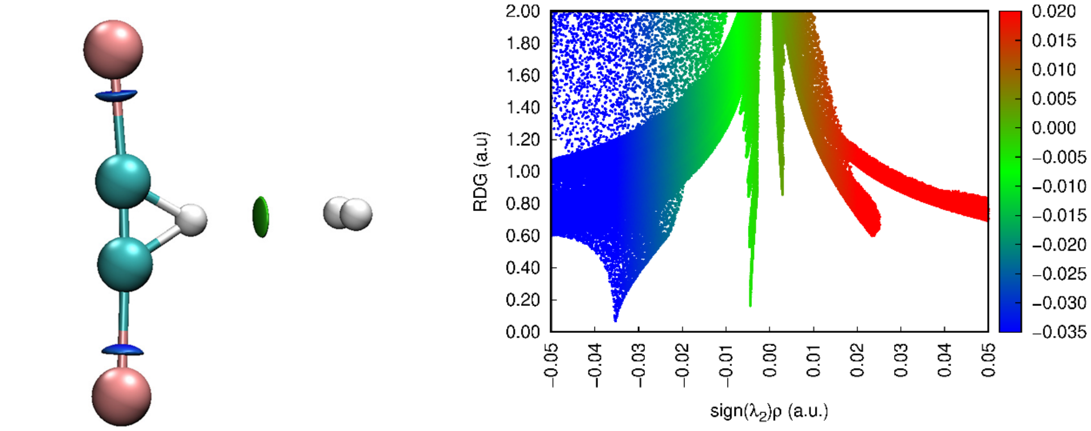
2.00
1.80
1.60
1.40
1.20
1.00
0.80
0.60
0.40
0.20
0.00
RDG (a.u)
−0.05
−0.04
−0.03
−0.02
−0.01
0.00
0.01
0.02
0.03
0.04
0.05
sign(λ2)ρ (a.u.)
0.020
0.015
0.010
0.005
0.000
−0.005
−0.010
−0.015
−0.020
−0.025
−0.030
−0.035

c

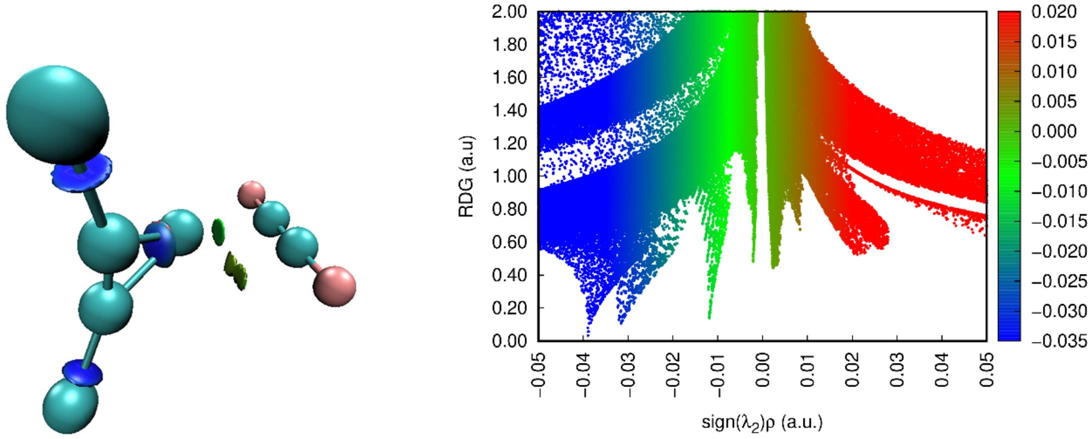
2.00
1.80
1.60
1.40
1.20
1.00
0.80
0.60
0.40
0.20
0.00
RDG (a.u)
−0.05
−0.04
−0.03
−0.02
−0.01
0.00
0.01
0.02
0.03
0.04
0.05
sign(λ2)ρ (a.u.)
0.020
0.015
0.010
0.005
0.000
−0.005
−0.010
−0.015
−0.020
−0.025
−0.030
−0.035

d

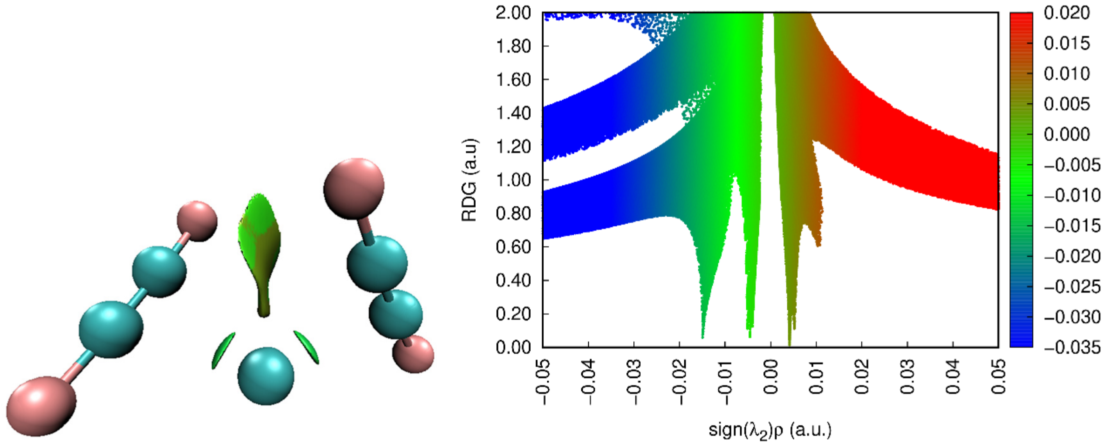
2.00
1.80
1.60
1.40
1.20
1.00
0.80
0.60
0.40
0.20
0.00
RDG (a.u)
−0.05
−0.04
−0.03
−0.02
−0.01
0.00
0.01
0.02
0.03
0.04
0.05
sign(λ2)ρ (a.u.)
0.020
0.015
0.010
0.005
0.000
−0.005
−0.010
−0.015
−0.020
−0.025
−0.030
−0.035

e

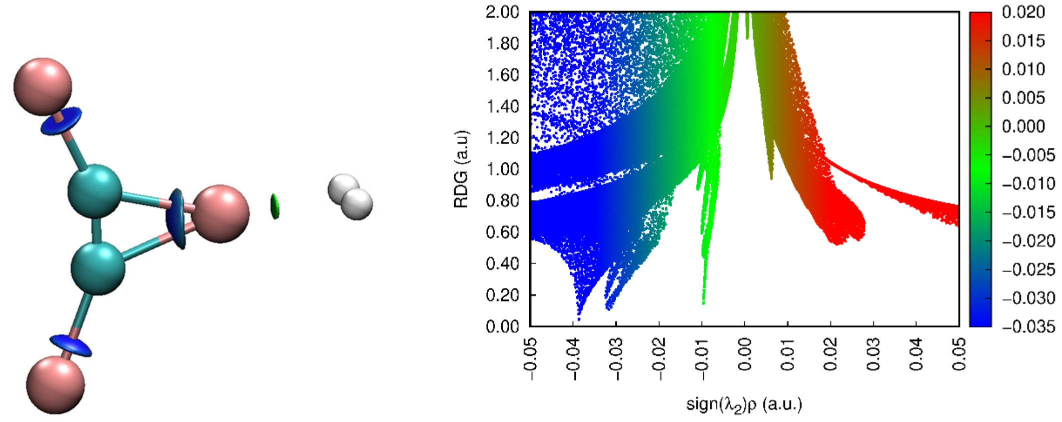
RDG (a.u)
sign(λ2)ρ (a.u.)
2.00
1.80
1.60
1.40
1.20
1.00
0.80
0.60
0.40
0.20
0.00
−0.05 −0.04 −0.03 −0.02 −0.01 0.00 0.01 0.02 0.03 0.04 0.05
0.020 0.015 0.010 0.005 0.000 −0.005 −0.010 −0.015 −0.020 −0.025 −0.030 −0.035

f

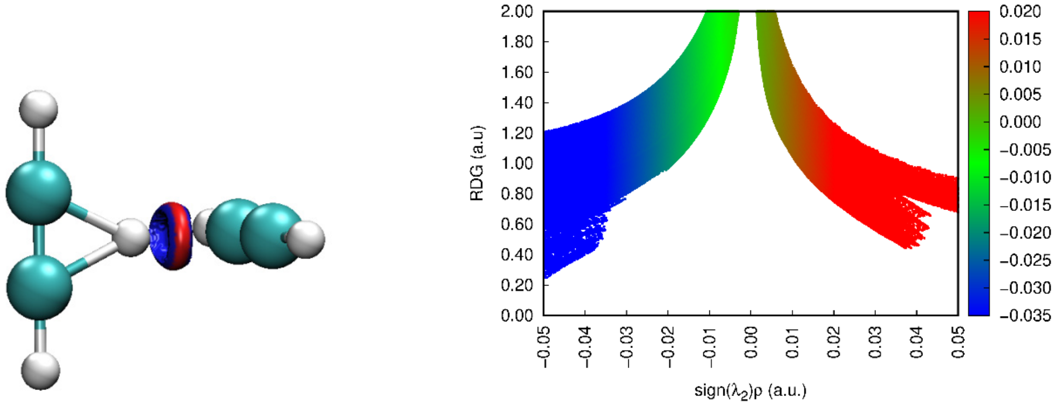
RDG (a.u)
sign(λ2)ρ (a.u.)
2.00
1.80
1.60
1.40
1.20
1.00
0.80
0.60
0.40
0.20
0.00
−0.05 −0.04 −0.03 −0.02 −0.01 0.00 0.01 0.02 0.03 0.04 0.05
0.020 0.015 0.010 0.005 0.000 −0.005 −0.010 −0.015 −0.020 −0.025 −0.030 −0.035

g

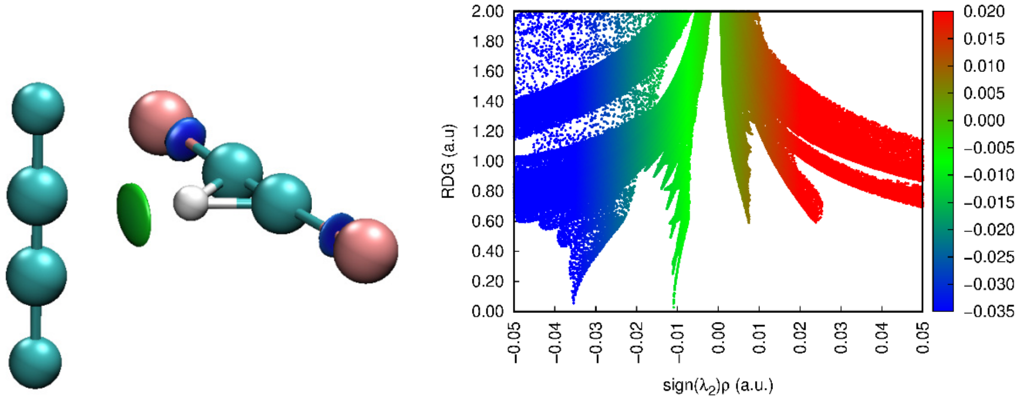
RDG (a.u)
sign(λ2)ρ (a.u.)
2.00
1.80
1.60
1.40
1.20
1.00
0.80
0.60
0.40
0.20
0.00
−0.05 −0.04 −0.03 −0.02 −0.01 0.00 0.01 0.02 0.03 0.04 0.05
0.020 0.015 0.010 0.005 0.000 −0.005 −0.010 −0.015 −0.020 −0.025 −0.030 −0.035

h

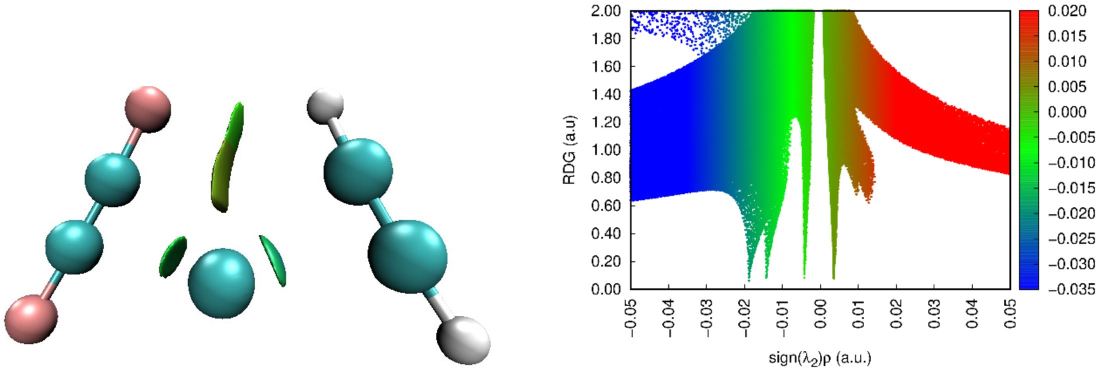
RDG (a.u)
sign(λ2)ρ (a.u.)
2.00
1.80
1.60
1.40
1.20
1.00
0.80
0.60
0.40
0.20
0.00
−0.05
−0.04
−0.03
−0.02
−0.01
0.00
0.01
0.02
0.03
0.04
0.05
0.020
0.015
0.010
0.005
0.000
−0.005
−0.010
−0.015
−0.020
−0.025
−0.030
−0.035
i

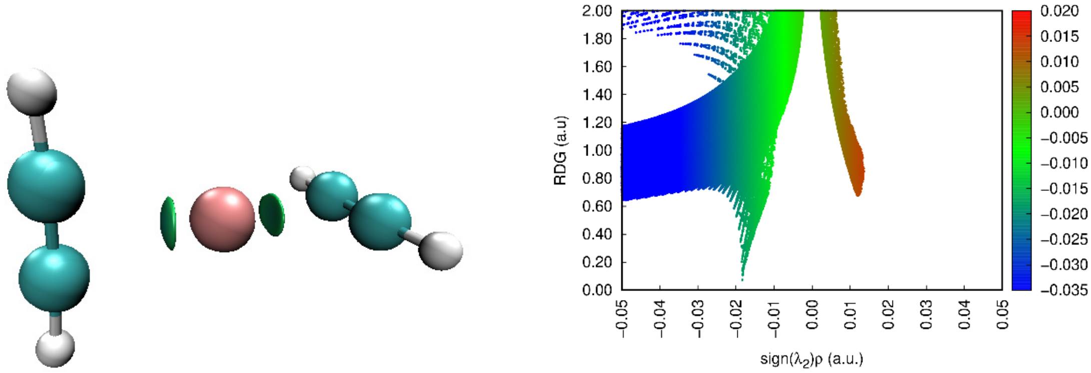
RDG (a.u)
sign(λ2)ρ (a.u.)
2.00
1.80
1.60
1.40
1.20
1.00
0.80
0.60
0.40
0.20
0.00
−0.05
−0.04
−0.03
−0.02
−0.01
0.00
0.01
0.02
0.03
0.04
0.05
0.020
0.015
0.010
0.005
0.000
−0.005
−0.010
−0.015
−0.020
−0.025
−0.030
−0.035
j

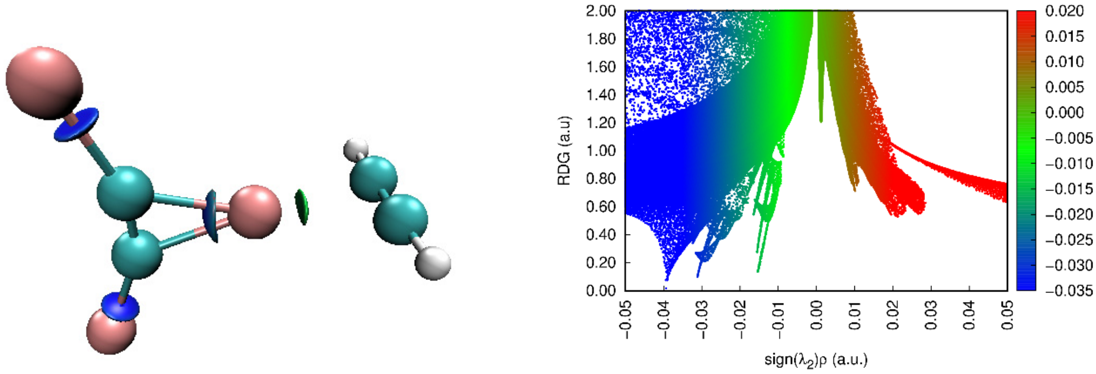
RDG (a.u)
sign(λ2)ρ (a.u.)
2.00
1.80
1.60
1.40
1.20
1.00
0.80
0.60
0.40
0.20
0.00
−0.05
−0.04
−0.03
−0.02
−0.01
0.00
0.01
0.02
0.03
0.04
0.05
0.020
0.015
0.010
0.005
0.000
−0.005
−0.010
−0.015
−0.020
−0.025
−0.030
−0.035
k

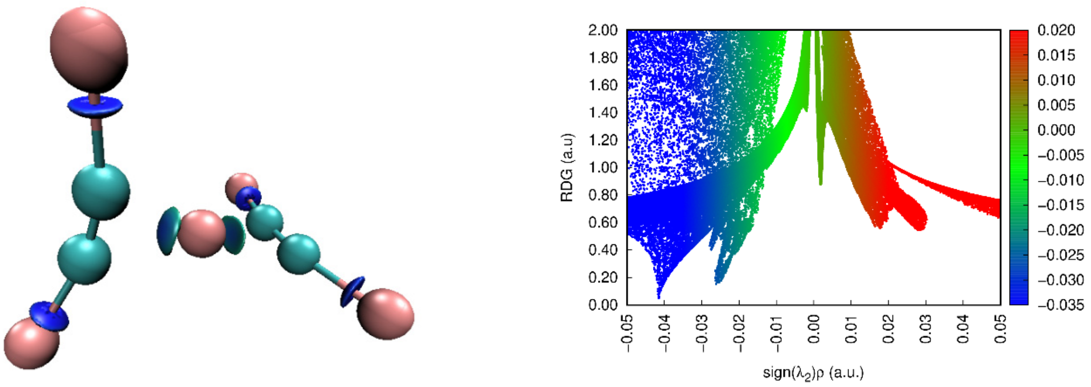
RDG (a.u)
sign(λ2)ρ (a.u.)
2.00 1.80 1.60 1.40 1.20 1.00 0.80 0.60 0.40 0.20 0.00
−0.05 −0.04 −0.03 −0.02 −0.01 0.00 0.01 0.02 0.03 0.04 0.05
0.020 0.015 0.010 0.005 0.000 −0.005 −0.010 −0.015 −0.020 −0.025 −0.030 −0.035

l

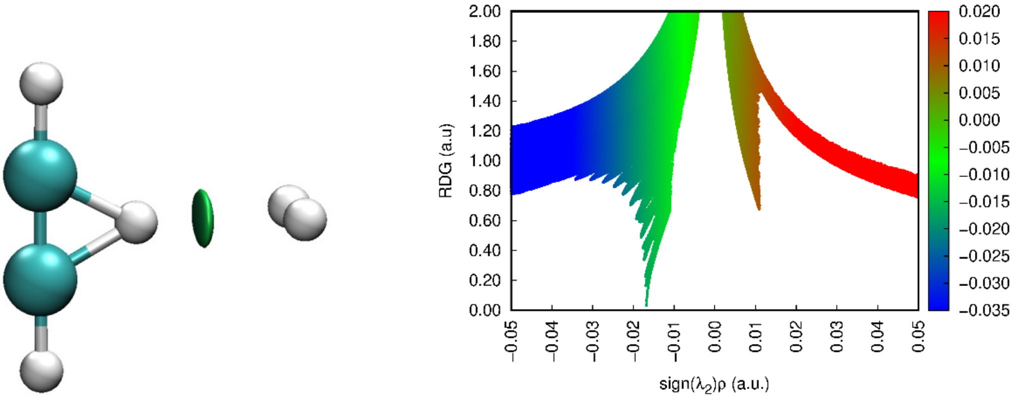
RDG (a.u)
sign(λ2)ρ (a.u.)
2.00 1.80 1.60 1.40 1.20 1.00 0.80 0.60 0.40 0.20 0.00
−0.05 −0.04 −0.03 −0.02 −0.01 0.00 0.01 0.02 0.03 0.04 0.05
0.020 0.015 0.010 0.005 0.000 −0.005 −0.010 −0.015 −0.020 −0.025 −0.030 −0.035

m

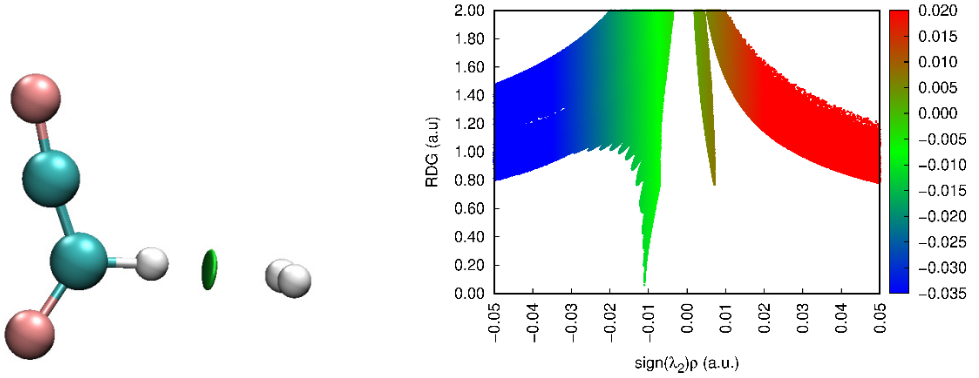
RDG (a.u)
sign(λ2)ρ (a.u.)
2.00 1.80 1.60 1.40 1.20 1.00 0.80 0.60 0.40 0.20 0.00
−0.05 −0.04 −0.03 −0.02 −0.01 0.00 0.01 0.02 0.03 0.04 0.05
0.020 0.015 0.010 0.005 0.000 −0.005 −0.010 −0.015 −0.020 −0.025 −0.030 −0.035

n

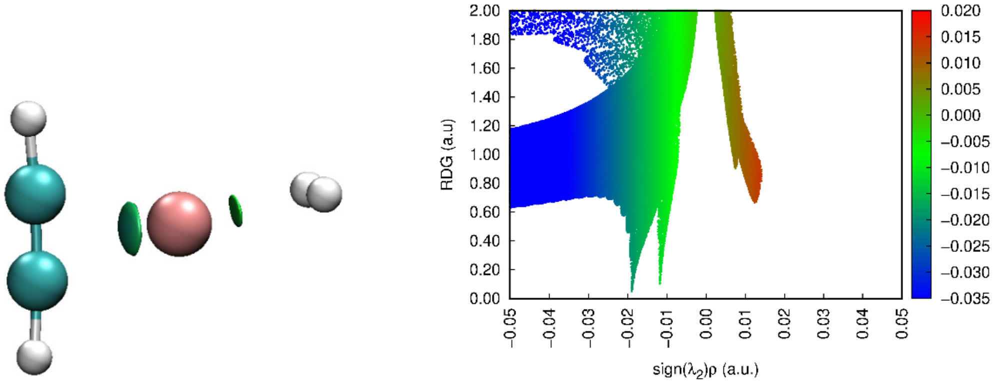

o

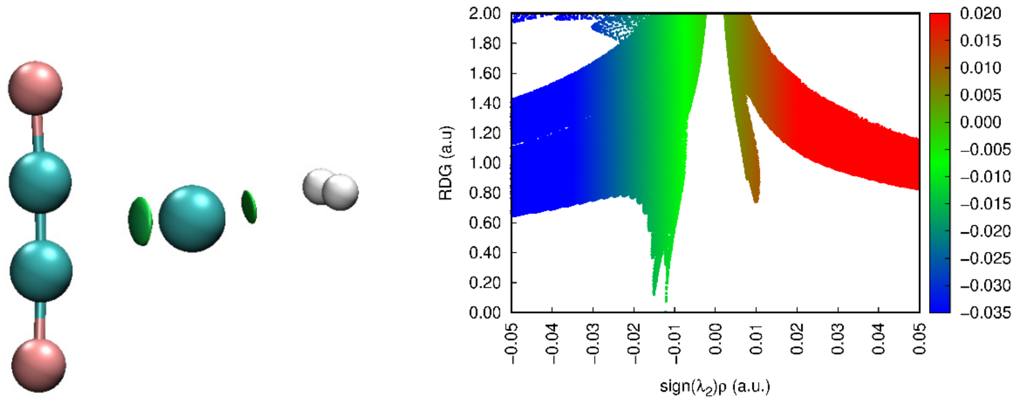

p

Figure S3 The RDG plots (right) and the corresponding molecular systems (left) of selected proton bound complexes. The following complexes are presented: (a) $C_2H_3^+$-C_2F_2, (b) $C_2F_2H^+$-C_2F_2, (c) $C_2Li_2H^+$-H_2, (d) $C_2Li_3^+$-C_2F_2, (e) $C_2F_2Li^+$-C_2F_2, (f) $C_2Li_3^+$-H_2. The sign(λ_2)ρ values between -0.1 and +0.1 a.u. The RDG value for surfaces presented at molecular structures is equal to 0.55.

These figures show that there are not additional weak interactions if the range of sign(λ_2)ρ is broader (in the main text sign(λ_2)ρ values between -0.05 and +0.05 a.u were applied); however the increase of RDG from 0.5 to 0.55 results in appearance of shapes not corresponding to interactions (they do not correspond to spikes).

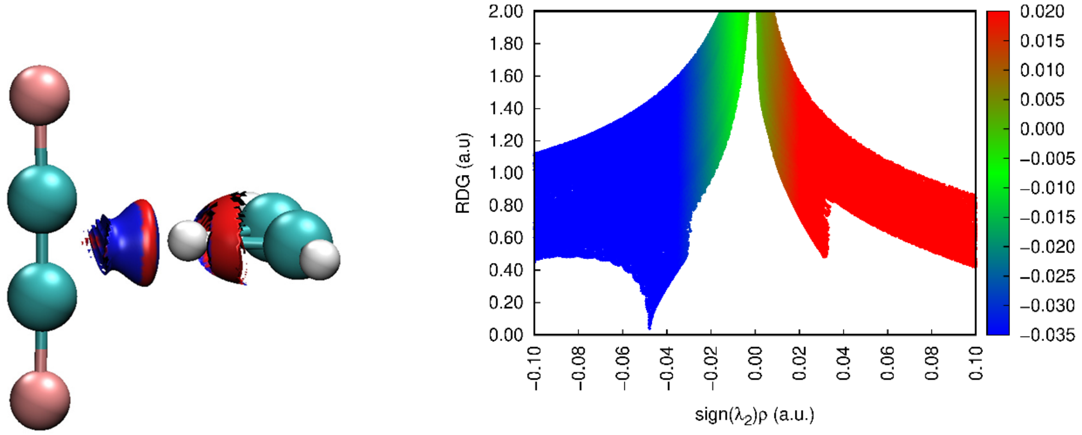
2.00
1.80
1.60
1.40
1.20
1.00
0.80
0.60
0.40
0.20
0.00
RDG (a.u)
−0.10
−0.08
−0.06
−0.04
−0.02
0.00
0.02
0.04
0.06
0.08
0.10
sign(λ2)ρ (a.u.)
0.020
0.015
0.010
0.005
0.000
−0.005
−0.010
−0.015
−0.020
−0.025
−0.030
−0.035

a

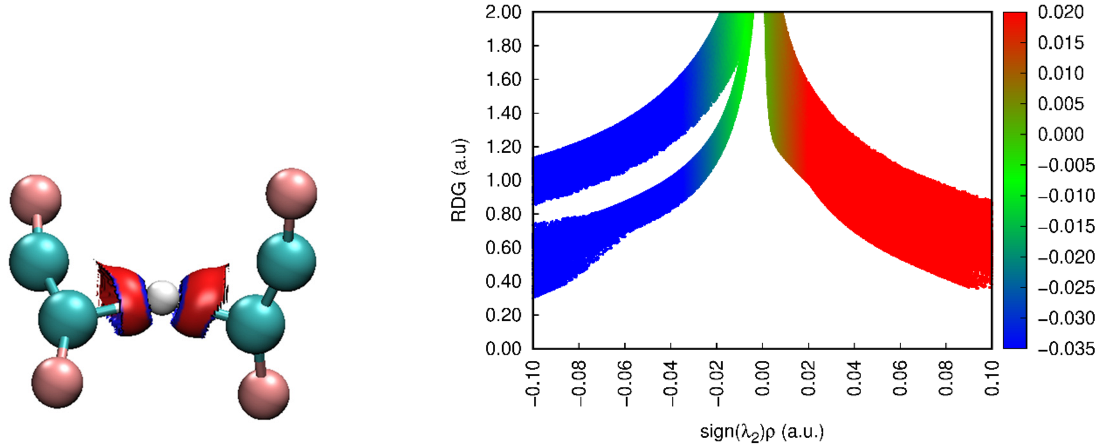
2.00
1.80
1.60
1.40
1.20
1.00
0.80
0.60
0.40
0.20
0.00
RDG (a.u)
−0.10
−0.08
−0.06
−0.04
−0.02
0.00
0.02
0.04
0.06
0.08
0.10
sign(λ2)ρ (a.u.)
0.020
0.015
0.010
0.005
0.000
−0.005
−0.010
−0.015
−0.020
−0.025
−0.030
−0.035

b

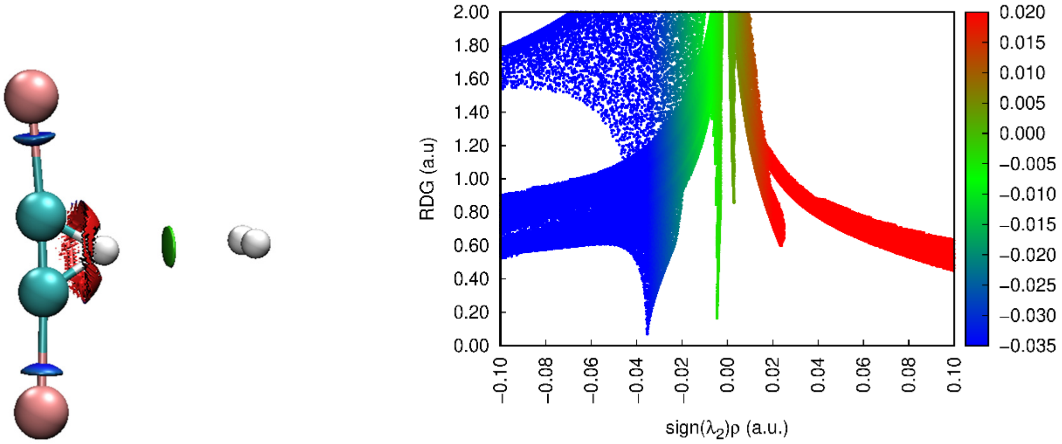
2.00
1.80
1.60
1.40
1.20
1.00
0.80
0.60
0.40
0.20
0.00
RDG (a.u)
−0.10
−0.08
−0.06
−0.04
−0.02
0.00
0.02
0.04
0.06
0.08
0.10
sign(λ2)ρ (a.u.)
0.020
0.015
0.010
0.005
0.000
−0.005
−0.010
−0.015
−0.020
−0.025
−0.030
−0.035

c

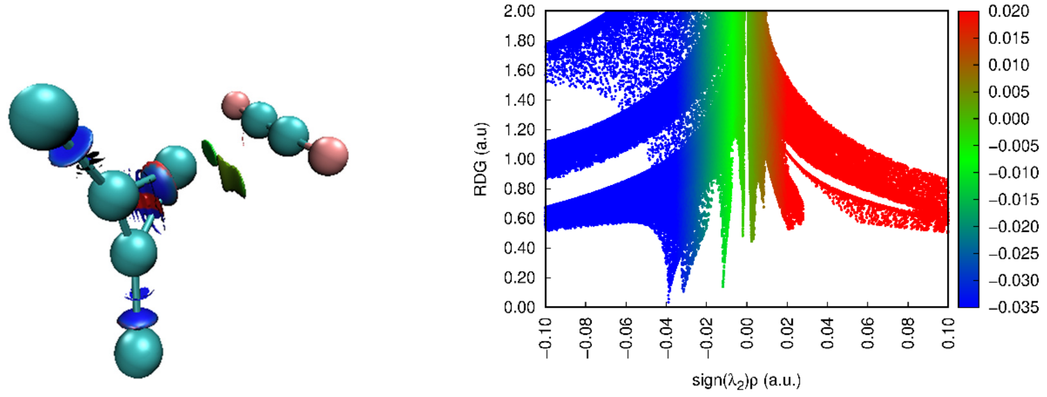
RDG (a.u)
sign(λ2)ρ (a.u.)
2.00
1.80
1.60
1.40
1.20
1.00
0.80
0.60
0.40
0.20
0.00
−0.10 −0.08 −0.06 −0.04 −0.02 0.00 0.02 0.04 0.06 0.08 0.10
0.020 0.015 0.010 0.005 0.000 −0.005 −0.010 −0.015 −0.020 −0.025 −0.030 −0.035

d

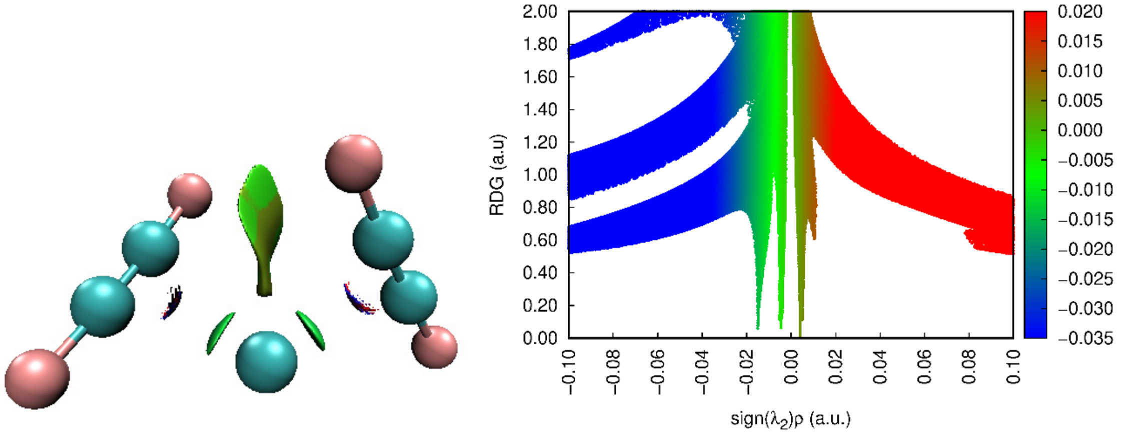
RDG (a.u)
sign(λ2)ρ (a.u.)
2.00
1.80
1.60
1.40
1.20
1.00
0.80
0.60
0.40
0.20
0.00
−0.10 −0.08 −0.06 −0.04 −0.02 0.00 0.02 0.04 0.06 0.08 0.10
0.020 0.015 0.010 0.005 0.000 −0.005 −0.010 −0.015 −0.020 −0.025 −0.030 −0.035

e

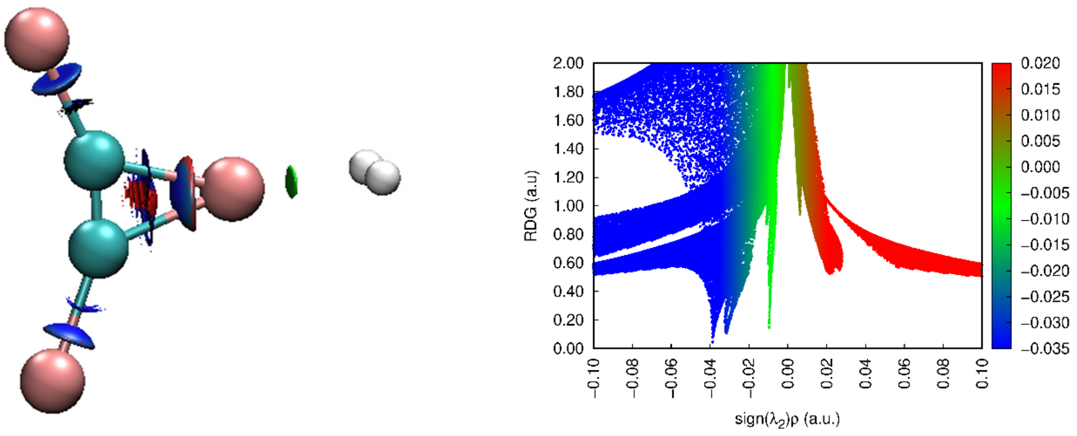
RDG (a.u)
sign(λ2)ρ (a.u.)
2.00
1.80
1.60
1.40
1.20
1.00
0.80
0.60
0.40
0.20
0.00
−0.10 −0.08 −0.06 −0.04 −0.02 0.00 0.02 0.04 0.06 0.08 0.10
0.020 0.015 0.010 0.005 0.000 −0.005 −0.010 −0.015 −0.020 −0.025 −0.030 −0.035

f

Table S1. The interaction energy terms (in kcal/mol) that are defined by equation 4 (main article); E_{int}^{HF}, is the Hartree-Fock interaction energy.

Complex	$E_{elst}^{(1)}$	$E_{exch}^{(1)}$	$E_{ind}^{(2)}$	$E_{dis}^{(2)}$	$E_{exch-ind}^{(2)}$	$E_{exch-di}^{(2)}$	δE_{HF}	E_{int}^{HF}	E_{int}^{SAPT2}
				Protonated systems					
$C_2H_3^+$-C_2H_2	-19.29	36.50	-27.63	-11.20	14.11	1.88	-13.93	-10.83	-19.56
$C_2H_3^+$-C_2F_2	-7.93	25.30	-21.38	-8.71	11.17	1.33	-8.98	-1.07	-9.19
$C_2F_2H^+$-C_2F_2	-15.12	64.96	-59.21	-17.18	29.11	1.62	-36.29	-15.12	-32.10
$C_2Li_2H^+$-C_2F_2	-1.52	4.24	-2.24	-3.29	1.40	0.43	-0.51	1.55	-1.48
$C_2H_3^+$-H_2	-2.21	4.78	-3.58	-1.78	1.19	0.21	-1.13	-0.79	-2.51
$C_2F_2H^+$-H_2	-1.33	2.35	-1.90	-0.98	0.52	0.10	-0.48	-0.92	-1.72
$C_2Li_2H^+$-H_2	-0.53	0.84	-0.28	-0.67	0.08	0.06	-0.08	0.03	-0.58
				Lithium species					
$C_2H_2Li^+$-C_2H_2	-13.08	7.75	-19.64	-1.04	8.55	0.12	-0.68	-18.98	-18.03
$C_2H_2Li^+$-C_2F_2	-2.44	5.31	-14.89	-1.75	5.54	0.14	-0.70	-7.31	-8.78
$C_2F_2Li^+$-C_2F_2	-2.66	6.17	-16.46	-2.42	6.25	0.20	-0.83	-7.75	-9.73
$C_2Li_3^+$-C_2H_2	-10.28	6.37	-12.42	-1.54	6.02	0.17	-0.68	-12.47	-12.36
$C_2Li_3^+$-C_2F_2	-2.43	4.63	-9.83	-1.89	4.60	0.23	-0.54	-3.78	-5.24
$C_2Li_3^+$-C_2Li_2	-53.69	19.70	-32.33	-3.44	19.39	0.62	-2.66	-52.04	-52.41
$C_2H_2Li^+$-H_2	-2.35	2.51	-6.96	-0.32	2.22	0.03	-0.20	-4.83	-5.07
$C_2F_2Li^+$-H_2	-2.42	2.61	-7.47	-0.29	2.36	0.03	-0.22	-5.22	-5.41
$C_2Li_3^+$-H_2	-1.94	1.98	-4.30	-0.44	1.49	0.04	-0.15	-2.99	-3.32
